# PUMA-induced apoptosis drives bone marrow failure and genomic instability in telomerase-deficient mice

**DOI:** 10.1038/s41418-025-01557-w

**Published:** 2025-08-19

**Authors:** Christian Molnar, Jovana Rajak, Julia Miriam Weiss, Irene Gonzalez-Menendez, Geoffroy Andrieux, Franziska Schreiber, Eva-Maria Kornemann, Lena Wendeburg, Gudrun Göhring, Brigitte Strahm, Fabian Beier, Doris Steinemann, Melanie Börries, Martina Rudelius, Leticia Quintanilla-Martinez, Charlotte M. Niemeyer, Marena R. Niewisch, Verena Labi, Sheila Bohler, Miriam Erlacher

**Affiliations:** 1 Department of Pediatrics and Adolescent Medicine, Division of Pediatric Hematology and Oncology, University Medical Center Freiburg, Faculty of Medicine, University of Freiburg, Freiburg, Germany; 2 Spemann Graduate School of Biology and Medicine (SGBM), University of Freiburg, Freiburg, Germany; 3 Faculty of Biology, University of Freiburg, Freiburg, Germany; 4 Department of Pediatrics and Adolescent Medicine, University Medical Center Ulm, Ulm, Germany; 5 Institute of Pathology and Neuropathology, Eberhard Karls University of Tübingen and Comprehensive Cancer Center, Tübingen, Germany; 6 University of Tübingen, Cluster of Excellence iFIT (EXC2180) “Image-Guided and Functionally Instructed Tumor Therapies”, Tübingen, Germany; 7 Institute of Medical Bioinformatics and Systems Medicine, Medical Center-University of Freiburg, Faculty of Medicine, University of Freiburg, Freiburg, Germany; 8 Institute of Pathology, Ludwig Maximilians University of Munich, Munich, Germany; 9 Department of Human Genetics, Hannover Medical School, Hannover, Germany; 10 Department of Hematology, Oncology, Hemostaseology and Stem Cell Transplantation, Medical Faculty, RWTH Aachen University, Aachen, Germany; 11 German Cancer Consortium (DKTK), Partner site Freiburg, a partnership between DKFZ and Medical Center - University of Freiburg, Freiburg, Germany; 12 Comprehensive Cancer Center Freiburg (CCCF), Medical Center, University of Freiburg, Faculty of Medicine, University of Freiburg, Freiburg, Germany; 13 Institute of Developmental Immunology, Biocenter, Medical University of Innsbruck, Innsbruck, Austria

**Keywords:** Paediatric cancer, Haematological diseases, Experimental models of disease

## Abstract

Bone marrow failure is a severe complication of human telomere biology disorders and predisposes individuals to secondary leukemia. A deeper understanding of this process could offer significant clinical benefits. Using a preclinical mouse model deficient in the RNA component of the telomerase (mTerc), we demonstrate that bone marrow failure results from excessive apoptosis, predominantly mediated by the pro-apoptotic p53 target PUMA. Genetic ablation of *Puma* alleviates hematological phenotypes and reduces the risk of lethal bone marrow failure while preserving genomic stability. Mechanistically, PUMA deficiency decreases the sensitivity of hematopoietic cells to lethal stressors, including critically short telomeres. As a consequence, reduced compensatory turnover of hematopoietic progenitors slows down telomere shortening at the population level, delays stem cell exhaustion, and diminishes the acquisition of somatic mutations - ultimately preventing neoplastic transformation. Elevated expression of both p53 and PUMA is also observed in the bone marrow from patients with telomere biology disorders. While apoptosis resistance is traditionally associated with malignant transformation, our findings provide evidence that selective inhibition of PUMA-mediated apoptosis may represent a viable therapeutic strategy to prevent or delay leukemic transformation in this patient population.

## Introduction

Inherited bone marrow failure syndromes (IBMFS), such as Fanconi anemia and dyskeratosis congenita, are rare disorders characterized by hematopoietic failure, extra-hematological manifestations, and cancer risk [1, 2]. Pancytopenia and bone marrow (BM) aplasia arise from progressive damage and depletion of hematopoietic stem and progenitor cells (HSPCs), often due to germline mutations affecting processes like DNA repair or telomere maintenance [3]. These defects impose proliferative and selective pressure on remaining HSPCs, fostering clonal hematopoiesis (CH) and promoting the development of secondary myelodysplastic syndrome (MDS) and acute myeloid leukemia (AML) [1, 4]. Preventing BM failure may thus not only restore hematopoiesis but also lower the risk for malignant transformation.

Telomere maintenance is critical for genome stability and cell longevity, relying on the coordinated activity of telomerase, shelterin proteins, and DNA repair pathways. Disruption of these systems result in telomere biology disorders (TBDs), defined by abnormally short telomeres and characterized by multisystem disease and cancer predisposition [3]. Mutations in at least 17 genes (e.g. *TERC, TERT, RTEL1, PARN, DKC1, TINF2)* underlie TBD. Although extra-hematological symptoms (e.g. liver/lung fibrosis) may occur, early mortality is primarily due to BM failure [5, 6], which can evolve into CH and MDS/AML [7–10].

TBD pathophysiology involves activation of ATR- and p53-dependend DNA-damage responses in cells with critically short telomeres [11–14]. Depending on the nature of the stress, p53 induces cell cycle inhibition, metabolic reprogramming, senescence, or apoptosis [15]. While these mechanisms eliminate damaged cells and maintain genomic integrity, they also contribute to HSPC attrition and BM failure [8, 15, 16]. Somatic *TP53* mutations that bypass these constraints are frequently observed in TBD patients and increase leukemic risk, as confirmed in mouse models [17–19].

We investigated whether blocking p53-mediated apoptosis – while preserving other p53 functions – could improve hematopoiesis in telomerase-deficient mice without compromising genomic stability. Therapeutically, prolonging the lifespan of hematopoietic cells may reduce compensatory proliferation, slow telomere shortening, and alleviate selective pressure, thereby preserving genome integrity [20]. Notably, irreparably damaged cells can still undergo p53-independent apoptosis, alternative cell death, or senescence [21].

In hematopoietic cells, PUMA – a BH3-only pro-apoptotic member of the BCL-2 family – is the major mediator of p53-induced apoptosis [22–24]. Under stress, BH3-only proteins inactivate anti-apoptotic BCL-2 proteins, enabling activation of the effector proteins BAX/BAK, mitochondrial permeabilization, and caspase activation [25]. Other pro-apoptotic proteins regulated by p53 include NOXA and BAX. PUMA is also regulated by p73 and FOXO3a and mediates apoptosis in response to DNA damage, ER stress, and oxidative stress [22–24, 26].

Sperka et al. provide early evidence that *Puma* deletion prolongs lifespan in telomerase-deficient mice [27]. Building on this, we analyzed the role of PUMA for hematopoietic failure in TBD. Using mice lacking the telomerase RNA component, mTerc, we show that PUMA-induced apoptosis substantially drives hematopoietic failure. *Puma* deletion markedly improves hematopoiesis and reduces risk of lethal BM failure, without compromising genomic stability. Elevated p53/PUMA levels in TBD patient BM suggest conservation of this pathway, underscoring PUMA as a potential therapeutic target for preventing BM failure and leukemic progression.

## Results

### PUMA-mediated apoptosis contributes to lethal BM failure in telomerase-deficient mice

**Fig. 1:**
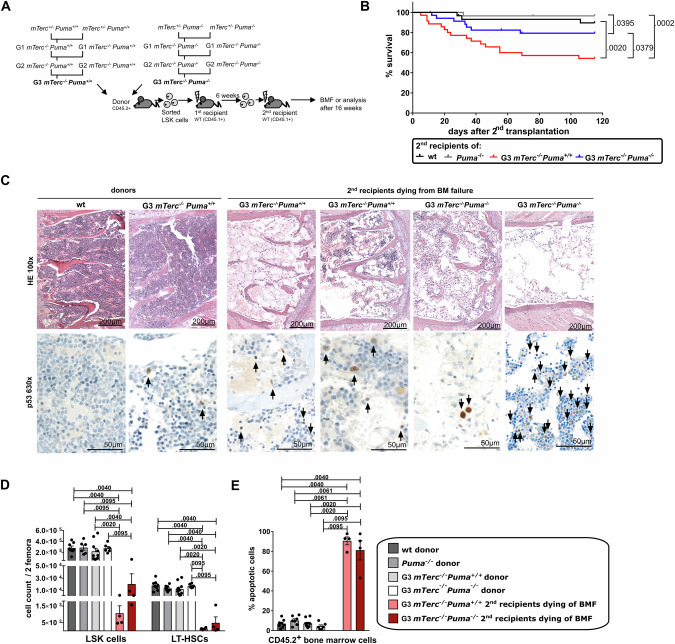
PUMA-deficiency reduces the risk of lethal BM failure in telomerase-deficient mice. **A** Third-generation (G3) telomerase-deficient mice (G3 *mTerc*^*-/-*^*Puma*^*+/+*^) and double-knockouts lacking telomerase and PUMA (G3 *mTerc*^*-/-*^*Puma*^*-/-*^) were generated to induce progressive telomere shortening. To further increase proliferative pressure and telomere shortening, G3 *mTerc*^*-/-*^*Puma*^*+/+*^and G3 *mTerc*^*-/-*^*Puma*^*-/-*^ LSK cells were serially transplanted into lethally irradiated CD45.1^+^ recipients. Secondary recipients were monitored for signs of BM failure or analyzed 16 weeks after secondary transplantation. **B** Survival of secondary recipients transplanted with LSK cells of the indicated genotypes. Some mice from both G3 *mTerc*^*-/-*^ groups succumbed to BM failure within 105 days. *n* = 30–35/group, ≥ 8 independent experiments, log-rank Mantel-Cox test. Mice with BM failure were analyzed by histopathology and flow cytometry, wildtype (WT), *Puma*^*-/-*^,G3 *mTerc*^*-/-*^*Puma*^*+/+*^ and G3 *mTerc*^*-/-*^*Puma*^*-/-*^ donors were included as controls. **C** Representative H&E staining (upper panels) and IHC for p53 (lower panels) were performed on sterna from wt and G3 *mTerc*^*-/-*^*Puma*^*+/+*^ donors and their respective secondary recipients with BM failure. Arrows indicate p53-positive cells. **D** BM cells of donors and secondary recipients that developed BM failure were used for flow cytometry. Cell counts of CD45.2^+^ LSK cells and long-term hematopoietic stem cells (LT-HSCs; LSK^+^CD48^-^CD150^+^) from 2 femora are shown (*n* = 4-10/group, ≥ 2 independent experiments). **E** BM cells were stained with CD45.1/2 antibodies, Annexin V and viability dye. CD45.2^+^ BM cells expressing Annexin V (with or without viability dye) were defined as % apoptotic cells (*n* = 4–10/group, ≥2 independent experiments). Bar graphs represent mean with SEM; a non-parametric Mann-Whitney test was used for statistical analysis.

We used mTerc-deficient mice (*mTerc*^*-/-*^) [28, 29] and generated double knockouts lacking both *mTerc* and PUMA (*mTerc*^*-/-*^*Puma*^*-/-*^). Owing to their longer telomeres (50–150 kB in mice vs. 5-15 kB in humans), *mTerc*^*-/-*^ mice initially showed no phenotype. Telomere shortening was induced by generation-breeding (Fig. 1A). G3 *mTerc*^*-/-*^ mice exhibited reduced body size and BM cellularity compared to WT and *Puma*^*-/-*^ mice (Supplementary Fig. 1A, B), but normal lineage distribution and marrow fat (Supplementary Fig. 1C). Peripheral blood showed isolated leukopenia, particularly lymphopenia (Supplementary Fig. 1D–G).

To induce BM failure, we transplanted LSK cells from G3 *mTerc*^*-/-*^*Puma*^*+/+*^ and G3 *mTerc*^*-/-*^*Puma*^*-/-*^ mice into CD45.1^+^ recipients. Consistent with previous reports [30], primary recipients showed no disease. However, a subset of secondary recipients succumbed within 105 days. Notably, G3 *mTerc*^*-/-*^*Puma*^*-/-*^ recipients had significantly better survival than PUMA-proficient controls (79.3% *vs*. 54.3%, *p* = 0.038; Fig. 1B). Deceased animals had severely hypoplastic BM with reductions in mature and progenitor cells, irrespective of donor genotype (Fig. 1C, D; Supplementary Fig. 1H). Immunohistochemistry confirmed presence of p53-positive cells (Fig. 1C), and Annexin V/Live Dead assays indicated extensive apoptosis (Fig. 1E). In contrast, WT or *Puma*^*-/-*^ secondary recipients remained healthy.

### Puma-deficiency improves hematopoiesis in G3 *mTerc*^*-/-*^ recipients

**Fig. 2:**
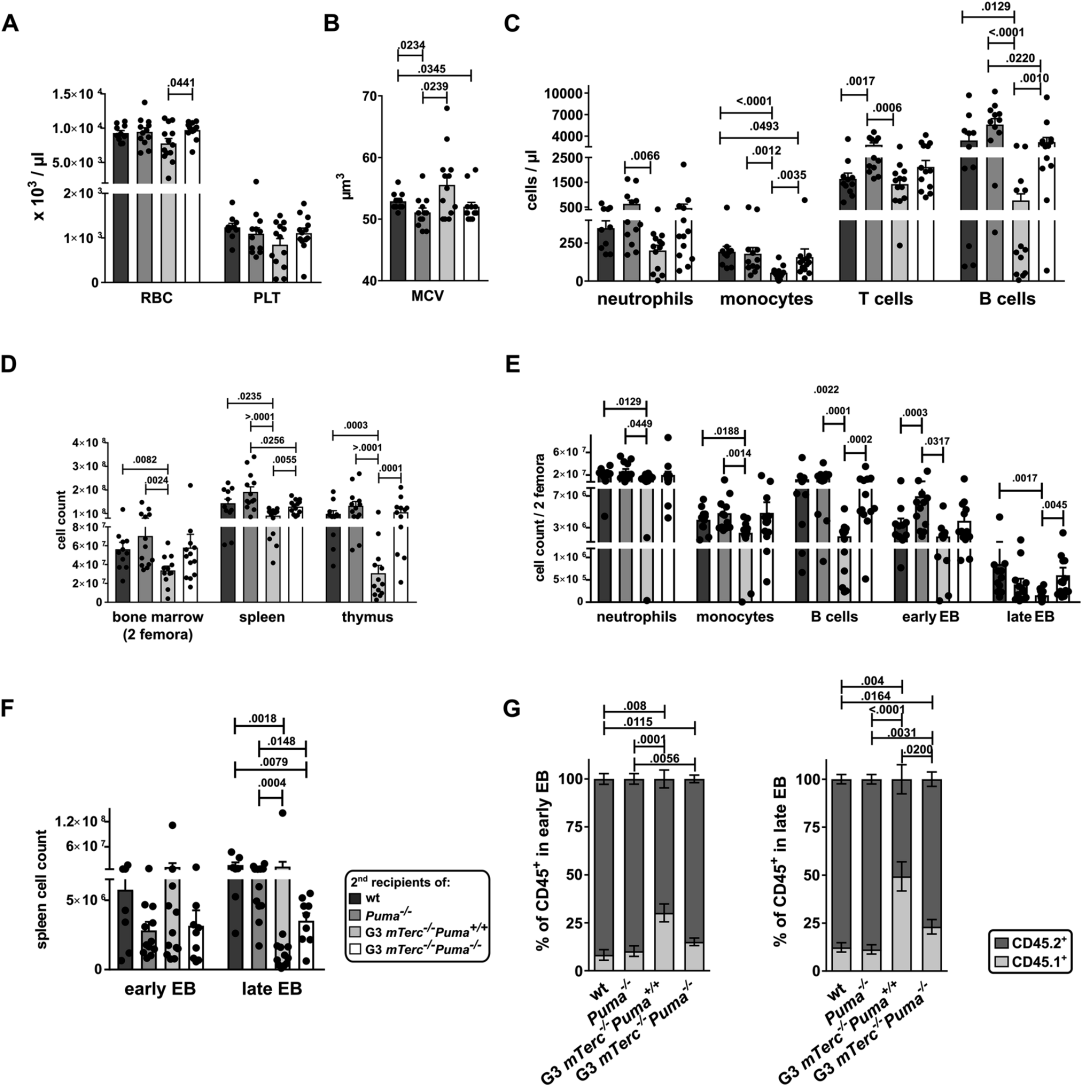
*Puma* deletion alleviates the hematological phenotype in the TBD mouse model. **A** Red blood cell (RBC) and platelet (PLT) counts as well as **B** mean corpuscular volume (MCV) of RBC are depicted. (*n* = 10-13/group, ≥ 3 independent experiments). **C** The indicated white blood cell (WBC) populations are shown: neutrophils = CD11b^+^Ly6G^+^, monocytes = CD11b^+^Ly6C^+^, T cells = TCR^+^, B cells = B220^+^ (*n* = 10-13/group, ≥ 3 independent experiments). **D** Total counts of BM (2 femora), spleen or thymus were evaluated for the indicated genotypes (*n* = 11–13/group, ≥3 independent experiments). **E** The indicated CD45.2^+^ cell populations in BM were evaluated by flow cytometry: early erythroblasts (EB) = CD71^+^Ter119^+^, late EB = CD71^-^Ter119^+^ (*n *= 11-13/group, 3–5 independent experiments). **F** Extramedullary erythropoiesis in spleen was determined by flow cytometry (early EB = CD71^+^Ter119^+^, late EB = CD71^-^Ter119^+^; *n* = 9–13, 3–5 independent experiments). **G** Ratio of CD45.1^+^ vs. CD45.2^+^ erythroid cells in spleen was determined by flow cytometry (*n* = 7–12, 2–4 independent experiments). All bar graphs show mean ± SEM; a non-parametric Mann-Whitney test was used.

**Fig. 3:**
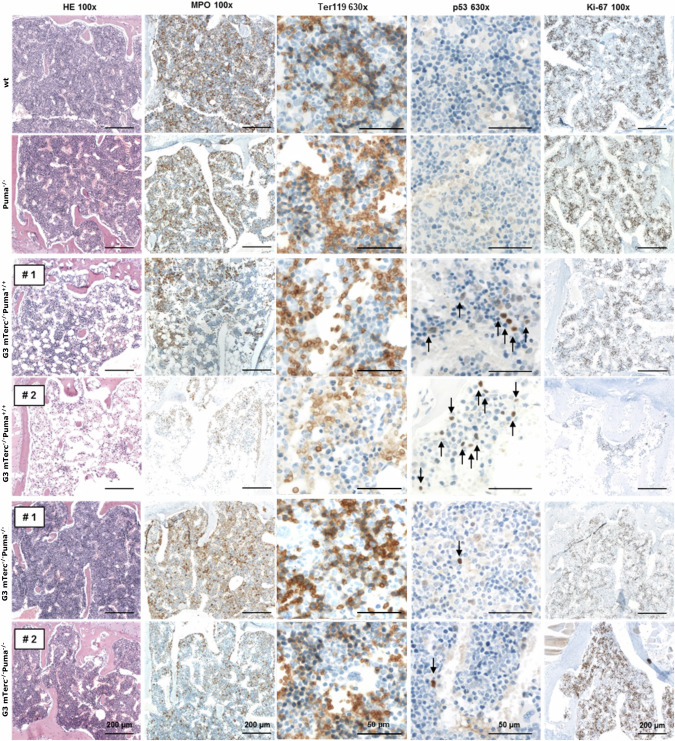
Hematological phenotype of secondary recipients of PUMA-proficient and deficient G3 *mTerc*^*-/-*^ mice. Secondary recipients were transplanted with LSK cells of the indicated genotype and sacrificed 16 weeks later. Sterna were fixed and embedded, and sections were stained with HE (left panel). Alternatively, immunohistochemistry was done for myeloid cells (MPO), erythroid cells (Ter119), p53 and Ki-67. Black arrows indicate p53-positive cells. In total, four secondary recipients of WT and *Puma*^*-*/-^ LSK cells, as well as six G3 *mTerc*^*-/-*^*Puma*^*+/+*^ and G3 *mTerc*^*-/-*^*Puma*^*-/-*^-transplanted secondary recipients from 3 independent experiments were analyzed. Representative pictures are shown.

Surviving secondary recipients were sacrificed 16 weeks post-transplantation. G3 *mTerc*^*-/-*^*Puma*^*+/+*^ recipients developed a phenotype resembling human TBD, with elevated mean corpuscular volume (MCV), mild anemia and thrombocytopenia as well as severe leukopenia (Fig. 2A–C, Supplementary Fig. 2). Cellularity of BM, spleen and thymus was significantly reduced compared to the recipients of other genotypes (Fig. 2D). BM histology and flow cytometry revealed hypoplasia and impairment across all hematopoietic lineages (Figs. 2E, 3; Supplementary Table 1). Extramedullary and recipient-derived erythropoiesis was elevated (Fig. 2F, G), indicating insufficient erythroid output from donor cells. In contrast, G3 *mTerc*^*-/-*^*Puma*^*-/-*^ recipients showed a phenotype largely comparable to WT controls, with only mild reductions in lymphocytes, erythroid progenitors, and spleen cellularity (Figs. 2, 3, Supplementary Table 1).

**Fig. 4:**
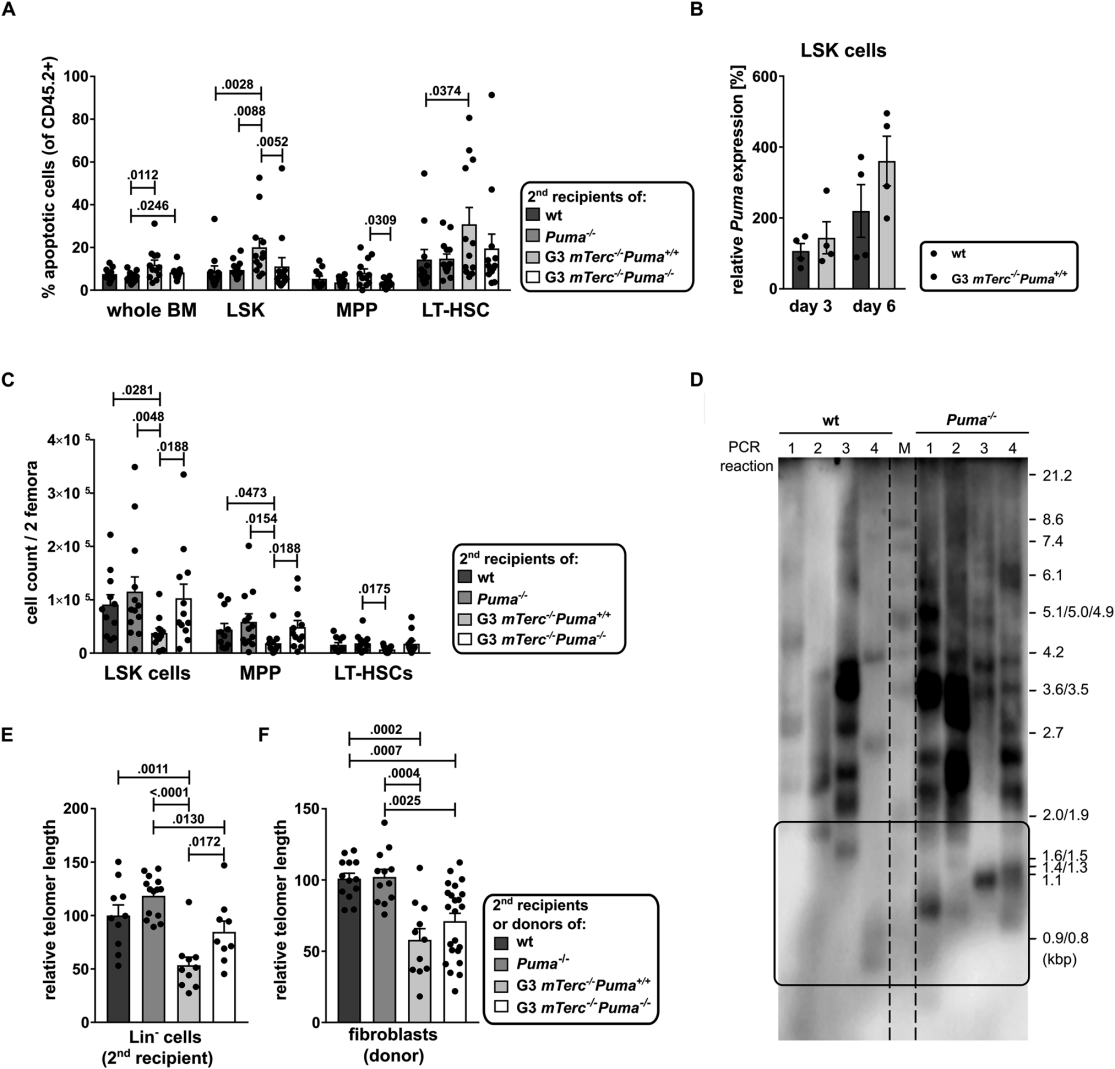
Deletion of *Puma* increases the number of functional stem cells and delays telomere shortening in the TBD mouse model. **A** Secondary recipients were transplanted with LSK cells of the indicated genotypes and sacrificed 16 weeks later. Apoptotic cells (Annexin V^+^) were determined within CD45.2^+^ total BM cells and HSPC populations by flow cytometry (*n* = 11–13/group, ≥3 independent experiments). Bars represent mean ± SEM; the non-parametric Mann-Whitney test was used for statistical analysis. **B** LSK cells were isolated from WT and G3 *mTerc*^*-/-*^*Puma*^*+/+*^ mice and cultivated in IMDM medium supplemented with 10% FCS and the cytokines SCF, TPO and FLT3-L (100 ng/µl each) to drive proliferation. On day 3 and 6 of in vitro culture, *Puma* mRNA levels were determined. Results were normalized to wt *Puma* expression on day 3 (*n* = 4/group, 1 independent experiment). Bars represent SEM; a non-parametric Mann-Whitney test was used. **C** Secondary recipients were transplanted with LSK cells of the indicated genotypes and sacrificed 16 weeks later. LSK cells, multipotent progenitors (MPP; LSK^+^CD48^+^CD150^-^) and long-term hematopoietic stem cells (LT-HSC; LSK^+^CD48^+^CD150^+^) from 2 femora are shown (*n* = 11–13/group, ≥ 3 independent experiments). **D** Telomere Shortest Length Assay (TESLA) performed on lineage-negative cells derived from bone marrow of wt and Puma^-/-^ mice reveals the presence of shorter telomeres in PUMA-deficient than proficient cells. **E** qPCR analysis for telomeric repeats was performed to determine telomere length (TEL) in lineage-marker negative (Lin^-^) BM cells. TEL was normalized to age-matched WT controls (*n* = 9-14/group; ≥ 3 independent experiments). **F** Fibroblasts derived from ear tissues of 8–12 weeks old mice were used for TEL measurement, WT fibroblasts of age-matched mice were used for normalization (*n* = 4–6/group, 3 independent experiments). Bars represent mean ± SEM, the non-parametric Mann-Whitney test was used for statistical analysis.

Apoptosis and p53-positive cells were elevated in BM of G3 *mTerc*^*-/-*^*Puma*^*+/+*^ recipients (Figs. 3, 4A). p53 expression was primarily detected in myeloid and erythroid cells (1-10%; Supplementary Fig. 3, Supplementary Table 1). These were markedly reduced in G3 *mTerc*^*-/-*^*Puma*^*-/-*^ recipients. To confirm PUMA as a direct apoptosis mediator in telomerase-deficient cells, we measured *Puma* mRNA in proliferating LSK cells from G3 *mTerc*^*-/-*^*Puma*^*+/+*^ and WT mice. While expression was higher in G3 *mTerc*^*-/-*^*Puma*^*+/+*^ LSK cells, the difference was not statistically significant (Fig. 4B).

### Stem cell functions are preserved in G3 *mTerc*^*-/-*^*Puma*^*-/-*^ mice and their recipients

We examined whether improved hematopoiesis in G3 *mTerc*^*-/-*^*Puma*^*-/-*^ mice compromised stem/ progenitor cell integrity. Sixteen weeks post-transplantation, G3 *mTerc*^*-/-*^*Puma*^*+/+*^ secondary recipients showed marked depletion of all HSPC populations compared to WT and *Puma*^*-/-*^ controls. No such depletion was found in G3 *mTerc*^*-/-*^*Puma*^*-/-*^ recipients (Fig. 4C).

To assess functional stem cell capacity, we transplanted 400 LT-HSCs from each genotype into lethally irradiated recipients, along with 200,000 CD45.1^+^ WT BM cells. G3 *mTerc*^*-/-*^*Puma*^*+/+*^ LT-HSCs failed to sustain hematopoiesis, especially lymphopoiesis (Supplementary Fig. 4). In contrast, G3 *mTerc*^*-/-*^*Puma*^*-/-*^ LT-HSCs efficiently supported multilineage reconstitution. These findings demonstrate that PUMA deficiency preserved both stem cell phenotype and function in telomerase-deficient mice.

### PUMA deficiency mitigates telomere erosion

To examine how PUMA loss affects telomere dynamics, we performed a “telomere shortest length assay” (TESLA) on WT and *Puma*^*-/-*^ splenocytes. *Puma*^*-/-*^ cells showed more very short telomeres than WT cells (Fig. 4D), suggesting survival of apoptosis-resistant cells despite critical telomere shortening.

To assess telomere shortening at the population level, we measured median telomere lengths in lineage-negative BM progenitors from secondary recipients using semi-quantitative qPCR. G3 *mTerc*^*-/-*^*Puma*^*-/-*^ cells had significantly longer telomeres than G3 *mTerc*^*-/-*^*Puma*^*+/+*^ counterparts (Fig. 4E). Telomerase-proficient *Puma*^*-/-*^ cells showed slightly longer telomeres than WT, and significantly longer telomeres than G3 *mTerc*^*-/-*^*Puma*^*-/-*^ cells (Fig. 4E). These results were corroborated by Southern blot of splenocytes from secondary recipients (Supplementary Fig. 5A). To assess potential transgenerational effects of systemic *Puma* loss, we analyzed fibroblasts from G3 *mTerc*^*-/-*^*Puma*^*-/-*^ and G3 *mTerc*^*-/-*^*Puma*^*+/+*^ mice. Both qRT-PCR and Southern blot revealed only a modest, non-significant telomere increase in PUMA-deficient fibroblasts (Fig. 4F, Supplementary Fig. 5B), indicating minimal transgenerational impact.

The combination of increased BM cellularity, reduced apoptosis, and longer median telomere lengths in G3 *mTerc*^*-/-*^*Puma*^*-/-*^ secondary recipients suggests delayed disease progression. Since proliferative stress drives telomere attrition and hematopoietic failure, we hypothesized that apoptosis resistance in *Puma*^*-/-*^ cells reduces HSPC turnover. Consistent with reduced turnover, we observed extended telomere lengths also in hematopoietic cells from donor *Puma*^*-/-*^ mice, with the most prominent effects seen in erythroid progenitors (Supplementary Fig. 6A). Erythropoietin levels were lower in *Puma*^*-/-*^ than in WT mice, while thrombopoietin levels remained unchanged (Supplementary Fig. 6B, C), further indicating reduced erythropoietic stress.

To assess proliferative pressure directly, we evaluated Ki-67 expression in BM of secondary recipients. All genotypes showed high Ki-67 levels, but some G3 *mTerc*^*-/-*^*Puma*^*+/+*^ recipients lacked Ki-67+ cells, suggesting hematopoietic exhaustion – a phenomenon not seen in G3 *mTerc*^*-/-*^*Puma*^*-/-*^ recipients (Fig. 3, Supplementary Table 1). No additional time point proved suitable for direct comparison of proliferative demand between genotypes.

### Genomic stability is preserved in G3 *mTerc*^*-/-*^*Puma*^*-/-*^ hematopoiesis

G1-G3 *mTerc*^*-/-*^*Puma*^*+/+*^ and G1-G3 *mTerc*^*-/-*^*Puma*^*-/-*^ donor and recipient mice did not developed any hematologic malignancies. However, the survival of cells with critically short telomeres poses a risk for genomic instability and transformation. We thus analyzed genomic integrity by multiple ways.

**Fig. 5:**
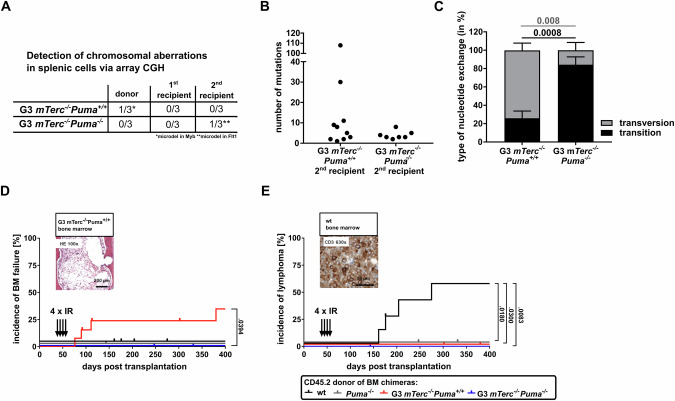
Genome stability is maintained in PUMA-deficient G3 *mTerc*^*-/-*^ mice. **A** Splenic cells from donors or their primary (6 weeks after transplantation) and secondary recipients (16 weeks after transplantation) were used for array-CGH to detect chromosomal aberrations. Only two small chromosomal deletions were detected (*n* = 2-3 /group). BM cells from secondary recipients of G3 *mTerc*^*-/-*^*Puma*^*+/+*^ and G3 *mTerc*^*-/-*^*Puma*^*-/-*^ were subjected to WES. For the germline control, ear tissue of donor mice was used. **B** Number of mutations is shown for each mouse (*n* = 7–10/group). **C** Proportion of nucleotide transversions and transitions is depicted (*n* = 7–10/group). Bars represent mean ± SEM, the non-parametric Mann-Whitney test was used. BM chimeras were subjected to 4 doses of 1.75 Gy (weekly) and monitored for lymphoma and leukemia development (*n* = 8–13/group, 2 independent experiments). **D** A small number of recipients transplanted with G3 *mTerc*^*-/-*^*Puma*^*+/+*^ BM cells succumbed due to BM failure, identified by HE staining of BM cross sections. **E** Incidence of lymphoma is shown (Kaplan-Meier blot; log-rank Mantel-Cox test). Representative CD3 IHC is shown.

To assess DNA double-strand breaks, we measured γH2AX levels in LSK cells 16 weeks post-secondary transplantation. Elevated γH2AX signals were found in 2 of 12 G3 *mTerc*^*-/-*^*Puma*^*+/+*^ samples, but none of the G3 *mTerc*^*-/-*^*Puma*^*-/-*^ samples (Supplementary Fig. 7A). Genomic integrity was analyzed using array-CGH and WES on splenocytes from donor and recipient mice. Array-CGH revealed no major chromosomal aberrations (Fig. 5A, Supplementary Fig. 8). However, WES revealed a high number of somatic mutations (variant allele frequency >5%) in 2 of 10 G3 *mTerc*^*-/-*^*Puma*^*+/+*^ secondary recipients, with a predominance of nucleotide transversions – hallmarks of genomic instability (Fig. 5B, C). Many mutated genes were functionally linked to p53 (Supplementary Fig. 9; Supplementary Table 2). In contrast, G3 *mTerc*^*-/-*^*Puma*^*-/-*^ secondary recipients exhibited few mutations and minimal transversions.

We evaluated whether *Puma* deletion promotes activation of alternative lengthening of telomeres (ALT), a mechanism linked to malignancy [31]. C-circle assays revealed only minor, non-significant ALT activity in immature cells from both G3 *mTerc*^*-/-*^*Puma*^*+/+*^ and G3 *mTerc*^*-/-*^*Puma*^*-/-*^ secondary recipients (Supplementary Fig. 7B). In forced proliferation assays, all LSK cells showed progressive telomere shortening and exhaustion (Supplementary Fig. 7C, D).

To test leukemogenic potential, we applied repeated sublethal irradiation. BM chimeras were generated to avoid systemic toxicity. In line with earlier work [32, 33], 50% of WT but none of *Puma*^*-/-*^ chimeras developed T-cell leukemia. Some irradiated G3 *mTerc*^*-/-*^*Puma*^*+/+*^ chimeras developed BM failure, reflecting impaired regenerative capacity. Strikingly, no leukemia or BM failure developed in G3 *mTerc*^*-/-*^*Puma*^*-/-*^ chimeras (Fig. 5D, E). Thus, *Puma* deletion conferred protection against genomic instability and malignant transformation in telomerase-deficient hematopoiesis.

### p53-induced pathways remain active in the absence of PUMA

**Fig. 6:**
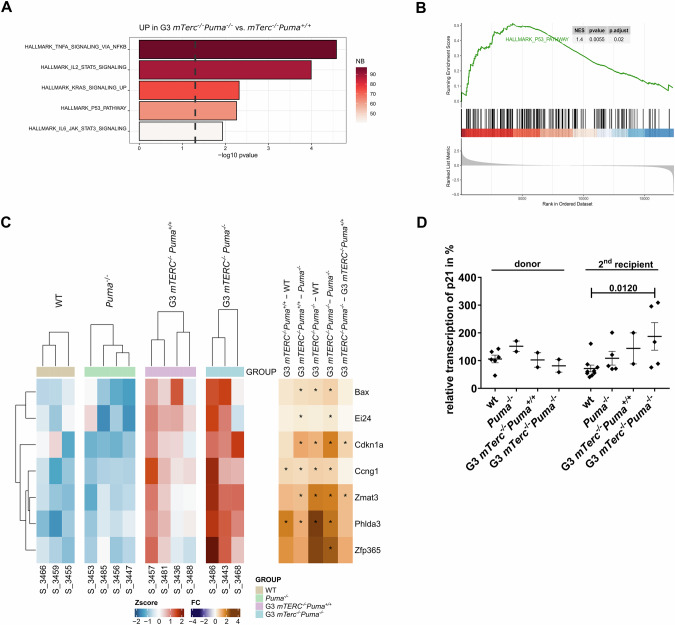
p53 signaling is maintained in the absence of PUMA. RNA was isolated from lineage-marker negative (Lin-) BM cells of secondary recipients of the indicated genotypes and subjected to RNAseq. Gene-set enrichment analysis suggests significantly enhanced pathways G3 *mTerc*^*-/-*^*Puma*^*-/-*^ as compared to G3 *mTerc*^*-/-*^*Puma*^*+/+*^ cells **A**, amongst other the HALLMARK_P53_Pathway **B**. **C** Expression of individual p53 target genes across all genotypes is shown in the heatmap, stars indicate significant differences. **D** qRT-PCR of p21/Cdkn1a was performed using mRNA isolated from LSK cells of donor and 2^nd^ recipient mice (*n* = 2–6; 2 independent experiments). The graph represents median ±SEM. P values were calculated with a non-parametric Mann-Whitney test.

To assess how PUMA deficiency affects downstream signaling in TBD, we performed RNA sequencing on lineage-negative progenitors from G3 *mTerc*^*-/-*^*Puma*^*+/+*^ and G3 *mTerc*^*-/-*^*Puma*^*-/-*^ secondary recipients. WT and *Puma*^*-/-*^ progenitors served as controls. Principal component analysis revealed genotype-dependent clustering (Supplementary Fig. 10A), and >250 differentially expressed genes between G3 *mTerc*^*-/-*^*Puma*^*+/+*^ and G3 *mTerc*^*-/-*^*Puma*^*-/-*^ cells. Gene set enrichment analysis showed upregulation of inflammatory and KRAS signaling pathways in G3 *mTerc*^*-/-*^*Puma*^*-/-*^ cells compared to their PUMA-proficient counterparts (Fig. 6A). p53 targets were also significantly enriched (Fig. 6B, C; Supplementary Fig. 10B), including *p21/Cdkn1a*, whose increased expression was confirmed by qRT-PCR (Fig. 6D). Together, these findings show that, in the absence of PUMA-mediated apoptosis, other p53-mediated tumor-suppressive functions remain active in telomerase-deficient hematopoiesis.

Compared to telomerase-proficient *Puma*^*-/-*^ cells, G3 *mTerc*^*-/-*^*Puma*^*-/-*^ cells showed enhanced signatures for cell cycle regulation, DNA damage checkpoints, senescence, and inflammation (Supplementary Fig. 10C), indicating persistent stress signaling despite absence of overt disease.

### PUMA is highly expressed in the hematopoietic system of TBD patients

**Fig. 7:**
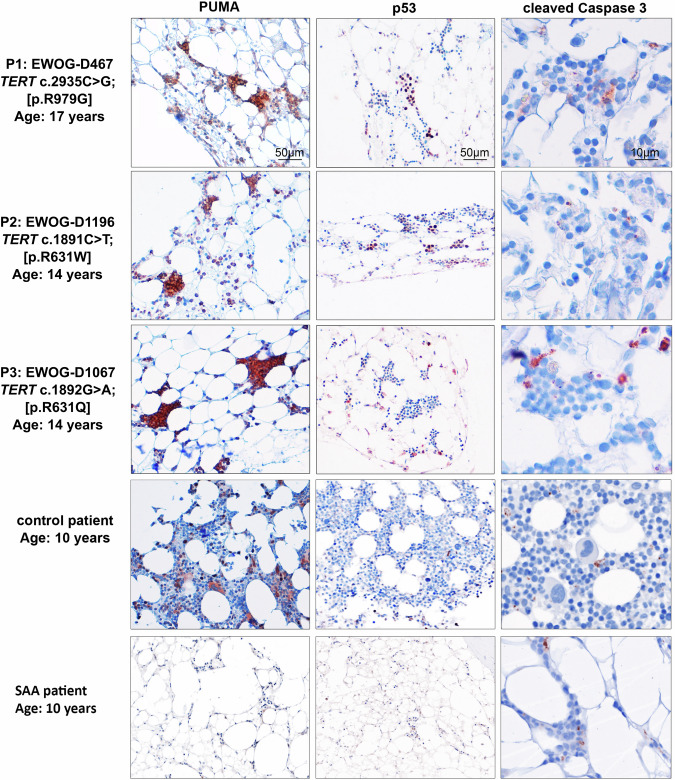
PUMA is highly expressed in bone marrow cells of TBD patients. Immunohistochemistry for PUMA (left), p53 (middle) and cleaved caspase 3 (right) was performed on bone marrow biopsies derived from three TBD patients (P1-3). The trephine biopsies of an age-matched control, who received BM analysis to exclude solid tumor infiltrations, and of a child with severe aplastic anemia were used as control. PUMA expression was especially abundant in erythroid precursors.

To assess clinical relevance, we analyzed PUMA and p53 expression in BM from three patients carrying *TERT* mutations (Supplementary Table 3). All showed pronounced BM aplasia and strong expression of both proteins across most hematopoietic cells – markedly higher than in an age-matched healthy control and a pediatric patient with severe aplastic anemia (Fig. 7; Supplementary Fig. 11), suggesting a conserved role of the p53/PUMA axis in TBD pathogenesis. Notably, only a small fraction of PUMA-positive cells co-stained for cleaved caspase-3, indicating limited active apoptosis (Fig. 7, right). This raises the possibility that somatic genetic or epigenetic events may have increased the apoptotic threshold, allowing survival despite pro-apoptotic signals.

## Discussion

Progressive HSPC depletion, leading to BM failure and cytopenias, is a hallmark of IBMFs. In TBD, Fanconi anemia and Shwachman-Diamond syndrome, compensatory proliferation of residual HSPCs increases cellular stress and accelerates disease. Although spontaneous somatic rescue events may restore hematopoiesis, they often carry leukemic risk [9, 34–42]. Introducing targeted “adaptive” mechanisms that preserve functional HSPCs could reduce proliferation and selection pressure, mitigating both BM failure and leukemic progression.

A central factor in these syndromes is p53, a key regulator of stress responses. While p53 inactivation poses oncogenic risks [16, 43–45], we demonstrate that selective inhibition of p53-mediated apoptosis, via *Puma* deletion, prevents BM failure without compromising genomic integrity in a TBD model. *Puma* deletion was previously shown to ameliorate the phenotype of *Mysm1*^*-/-*^ and NUP98-HOXD13 positive mice – two models in which cytopenias arise from excessive p53 activation [46, 47]. We now identify TBD as another context where PUMA-induced apoptosis is a key disease driver. Furthermore, we provide a direct mechanistic link between critical telomere shortening and PUMA, demonstrating that *Puma*^*-/-*^ cells tolerate shorter telomeres better than WT cells. Notably, *Puma*^*-/-*^ mice show no hematological abnormalities under homeostasis, highlighting that PUMA is dispensable in steady-state but essential under stress.

In our model, apoptosis affected both progenitor and mature BM compartments –broader than in other TBD models, where it was mainly restricted to mature cells [48]. Although cell turnover couldn’t be directly measured, longer telomeres and reduced cell loss in PUMA-deficient animals suggest reduced proliferative pressure and preserved HSPC function. Lifespan studies of specific hematopoietic populations may clarify this further.

Several limitations warrant consideration. First, our breeding design cannot entirely rule out transgenerational or microenvironmental effects of PUMA deficiency, such as protective influences on germ cells. An alternative approach, e.g. generational breeding of *mTerc*^*-/-*^*Puma*^*+/-*^ mice or conditional *Puma* deletion, would refine the model. Nonetheless, comparable telomere lengths in ear tissue of G3 *mTerc*^*-/-*^*Puma*^*+/+*^ and of G3 *mTerc*^*-/-*^*Puma*^*-/-*^ mice suggest minimal experimental bias. Second, *Puma* deletion had limited impact on lymphopoiesis, implying roles for other BH3-only proteins – particularly BIM or BMF [49, 50] – or a myeloid bias in *mTerc*^*-/-*^ HSPCs [48]. Finally, some secondary G3 *mTerc*^*-/-*^*Puma*^*-/-*^ recipients still developed BM failure with extensive cell death, suggesting contributions from p53-independent death pathways (i.e. extrinsic apoptosis, pyroptosis, necroptosis) or HSPC senescence – possibly driven by inflammation [51–53].

Our results challenge the dogma of apoptosis as universally tumor-suppressive [54]. While apoptosis resistance facilitates transformation in many contexts [55, 56], under chronic stress it may protect from genomic instability. Indeed, PUMA-deficient cells maintained genomic integrity and retained intact DNA damage responses. Importantly, these cells did not acquire p53-associated mutations, suggesting that apoptosis inhibition limited maladaptive clonal evolution.

Prior studies showed that p21 deletion also improves hematopoiesis in telomerase-deficient mice [57]. Future work should systematically compare inhibition of different p53 outputs – apoptosis, senescence, cell cycle arrest – to identify optimal targets. Dual pathway inhibition may offer additive benefits but could increase genomic instability [58].

Regarding therapeutic PUMA inhibition, timing is key. Early intervention – when cytopenias emerge but before malignant clones expand – is likely most effective. Later treatment could be harmful, especially in the presence of oncogenic mutations [59]. Inducible *Puma* deletion or pharmacological inhibition at defined stages will clarify safe time windows. Potential effects of PUMA inhibition on non-hematopoietic tissues also warrant study. Notably, PUMA loss protected intestinal cells in telomerase-deficient mice [27].

Broader apoptosis inhibition (e.g. BCL-2/BCL-XL overexpression) may also raise apoptotic thresholds [50, 60], but PUMA is an attractive target due to its stress-specific function and lack of known human loss-of-function syndromes [23, 25]. We are convinced that our findings can complement emerging therapeutic approaches like in vitro telomere elongation (ClinicalTrials.gov NCT04211714).

In summary, we identify PUMA-mediated apoptosis as a modifiable driver of BM failure and leukemic risk in TBD. Inhibiting PUMA promotes survival of stressed hematopoietic cells, prolongs blood formation, and delays disease progression. We propose this strategy as a therapeutic strategy for TBD.

## Methods

### Mice

*mTerc*^*-/-*^ and *Puma*^*-/-*^ mice [22, 28, 29] were inbred to generate Generation 3 (G3) offspring. All mice were on a CD45.2 C57BL/6 J background and maintained under specific-pathogen-free conditions. CD45.1 expressing B6.SJL-Ptpr^a^Pepc^b^/BoyJ mice served as recipients. All animal procedures were performed in accordance with the good scientific conduct guidelines and regulations of the ethics committee of the University of Freiburg as well as the local authorities - Regierungspräsidium Freiburg with registration number G15/09 and G19/135.

### Patient samples

BM biopsies from three patients with *TERT* mutation were provided by the EWOG-MDS consortium. One age-matched healthy individual and one patient with severe aplastic anemia served as controls. Informed consent and ethics approval was obtained from the local committee (no 247/05 and no 127/14).

### BM failure model

LSK (Lin^-^Sca^+^cKit^+^) cells were isolated from 8-12-week-old donors and transplanted into lethally irradiated (9.5 Gy) CD45.1^+^ recipients (20,000 LSK/mouse). After six week, 25,000 CD45.2^+^ LSK cells were re-transplanted into secondary recipients. Mice were sacrificed upon disease signs or after 16 weeks.

### LT-HSC transplantation

Lethally irradiated CD45.1^+^/CD45.2^+^ recipients were reconstituted with 400 LT-HSCs (CD48^-^150^+^ LSK) and 200,000 CD45.1^+^ BM cells and monitored for one year.

### Irradiation-induced lymphomagenesis

BM chimeras were generated by transplanting 2,000,000 CD45.2^+^ BM cells. Six weeks later, recipients received 4 weekly doses of 1.75 Gy [32, 33]. Mice were monitored for signs of disease.

### Cell counts and flow cytometry

Blood was analyzed via VetAbc (ScilAnimalCare) and hemocytometer. BM was collected from tibias, femora and pelvis; cell numbers were calculated for 2 femora. Blood, BM and splenocytes were subjected to RBC lysis and stained with antibodies (Supplementary Table 4). Dead cells were excluded using the Fixable Viability Dye eFluor™ 506 (ThermoFisher). Apoptosis was determined by AnnexinV (BioLegend) and eFluor™ 506 or 7-AAD (Sigma-Aldrich). Data were acquired on a BD LSRFortessa and analyzed with FlowJo (TreeStar Inc). Gating strategies are shown in Supplementary Fig. 12.

### Histopathology

Murine sterna were fixed in 4% paraformaldehyde, decalcified in EDTA, and paraffin embedded. Sections were stained with H&E or professed for immunohistochemistry using an automated system (Ventana Medical Systems). Antibodies are listed in Supplementary Table 4. Images were acquired with an Axioskop 2 plus Zeiss microscope and processed with ImageAccess and Photoshop (Adobe). Human BM biopsies underwent antigen retrieval (DAKO ph9 or ph6), immunostaining (Supplementary Table 4) and detection via the DAKO REAL^TM^ system. PUMA-positive cells were counted in 3 non-overlapping high-power fields (630x).

### LSK cell isolation and culture

After MACS depletion of Lin^+^ cells, Sca-1^+^cKit^+^ cells or LSK^+^CD48^-^150^+^ LT-HSC were FACS-sorted. LSK cells were cultured in 96-well plates with IMDM supplemented with 10% FCS and penicillin/streptomycin (ThermoFisher), SCF, TPO and FLT3-L (100 ng/ml each, ImmunoTools).

### Median telomere length determination

gDNA was extracted using QCP lysis [61] or the DNeasy® Blood & Tissue kit (Qiagen). Telomere qPCR was performed in triplicates using SYBR Green Mix (ThermoFisher) on a Roche LightCycler 480 (Supplementary Table 5). Relative telomere lengths were calculated by the 2^−ΔΔCt^ method using *36B4* as reference gene. ΔCt values were normalized to WT controls on each plate.

### Southern blot

DNA was extracted and digested with HinfI and RsaI. Fragments were separated on 0.3% agarose gels (36 h, 25 V), transferred to nylon membranes, and crosslinked by UV. After prehybridization, membranes were incubated overnight at 42 °C with a DIG-labeled telomeric probe. Post-hybridization washes were followed by incubation with anti-DIG antibody (1:10.000), and chemiluminescent detection was performed using CDP-STAR. Images were captured using Fusion FX (Vilber Lourmat).

### Telomere shortest length assay

TESLA was performed as described by Lai [62]. gDNA was ligated to TESLA-T oligos (Supplementary Table 5), digested to create short AT/TA overhangs, and ligated to partially double-stranded adaptors. PCR amplification of terminal fragments was performed using adaptor- and oligo-specific primers. Products were separated on 0.8% agarose gels, transferred to nylon membranes, and hybridized with a DIG-labeled telomere probe as described above.

### C-circle assay

ALT activity was assessed using the c-circle assay of Henson [63]. Circular telomeric DNA was amplified using Φ29 polymerase (NEB), followed by telomere qPCR. U-2OS osteosarcoma cells served as ALT-positive control. ALT activity was quantified by comparing qPCR signals with and without c-circle amplification.

### RNAseq

RNA was extracted from lineage-negative BM cells (RNeasy Micro kit, Qiagen). Libraries were prepared using the TruSeq Stranded mRNA Kit (Illumina), starting from ≤500 ng total RNA. After poly(A) selection, RNA was fragmented, reverse transcribed, and amplified (15 cycles). Libraries were validated (TapeStation, Qubit) and sequenced on NovaSeq 6000 (PE199, 399 pm with 1% PhiX). Quality control used FastQC (v0.11.9) and RSeQC (v4.0.0). Trimmomatic was used to trim adapters (HEADCROP:3 TRAILING:10 MINLEN:25) [64]. Read-per-gene was quantified using STAR aligner (v2.5.2b):--outFilterMultimapNmax 10 --alignSJoverhangMin 8 --alignSJDBoverhangMin 1 --outFilterMismatchNmax 999 --outFilterMismatchNoverLmax 0.05 i--alignIntronMin 20 --alignIntronMax 1000000 --alignMatesGapMax 1000000 --outFilterMatchNmin 16 [65]. GRCm38 was used to build the STAR index. A median of 110 million reads were uniquely mapped to the reference genome. Differential gene expression was analyzed using *limma* (v3.54.2) [65], and pathway enrichment with *clusterProfiler* (v4.6.2) using MSigDB gene-sets [39, 66]. Adjusted *p* < 0.05 was considered significant.

### Quantitative reverse transcription PCR

RNA was extracted using the MicroSpin Total RNA kit (VWR). cDNA was synthesized with the QuantiTect® Reverse Transcription Kit (Qiagen). qRT-PCR was performed using >25 ng cDNA. *GAPDH* served as housekeeping gene. Primers are listed in Supplementary Table 6.

### Array-CGH

gDNA from CD45.2^+^ splenocytes was extracted (DNeasy®, Qiagen) and hybridized to Agilent Mouse Genome 4x180k arrays. Slides were scanned (2 µm resolution), and data analyzed using Feature Extraction and Genomic Workbench (Agilent). Aberrations were called using the ADM2 algorithm (threshold 6.0, ≥ 4 probes, log2 ratio of -0.3).

### WES

gDNA quality was assessed by NanoDrop and Bioanalyzer. Libraries were prepared using Agilent SureSelectXT Mouse All Exon Kit and validated (TapeStation, Qubit). Sequencing was performed on a NovaSeq 6000 (PE100, 400 pM, 1% PhiX). Reads were trimmed (Trimmomatic) [64] and aligned to mm10 (mouse) or hg19 (human) using BWA-MEM. Post-processing (GATK) included realignment and base recalibration. Variant calling was done with Varscan2; annotation used ANNOVAR [67, 68]. Synonymous/non-exonic variants were excluded. Rare human variants were filtered via gnomAD (MAF < 0.001 or unknown) [69], and pathogenicity predicted by CADD13_PHRED > 20, DANN > 0.96, and Condel = Deleterious [70–72]. Mouse variants were annotated using GeneAnswers. Variants with <50 reads were excluded.

### Statistics

No data were excluded. Statistical tests included Mann-Whitney U for group comparisons, long-rank for survival (Kaplan-Meier), and the two-way ANOVA with Tukey’s post-hoc test for time-course data. Significance was defined as *P* < 0.05. Analyses were performed using Graphpad Prism.

## Supplementary information

Supplemental material

Supplemental table 2

uncropped blots

## Data Availability

The next-generation sequencing data is deposited at NCBI GEO (GSE299229). All other data generated and analyzed during this study are included in this published article and supplementary information.
