# Supplementary material for: PUMA-induced apoptosis drives bone marrow failure and genomic instability in telomerase-deficient mice
Source: Cell Death Differ. 2025 Aug 19;33(1):38–50. doi: 10.1038/s41418-025-01557-w (PMC12811261; doi:10.1038/s41418-025-01557-w)
Supplement: Supplementary file 1 — Supplemental material [file 41418_2025_1557_MOESM1_ESM.pptx]

## Slide 1
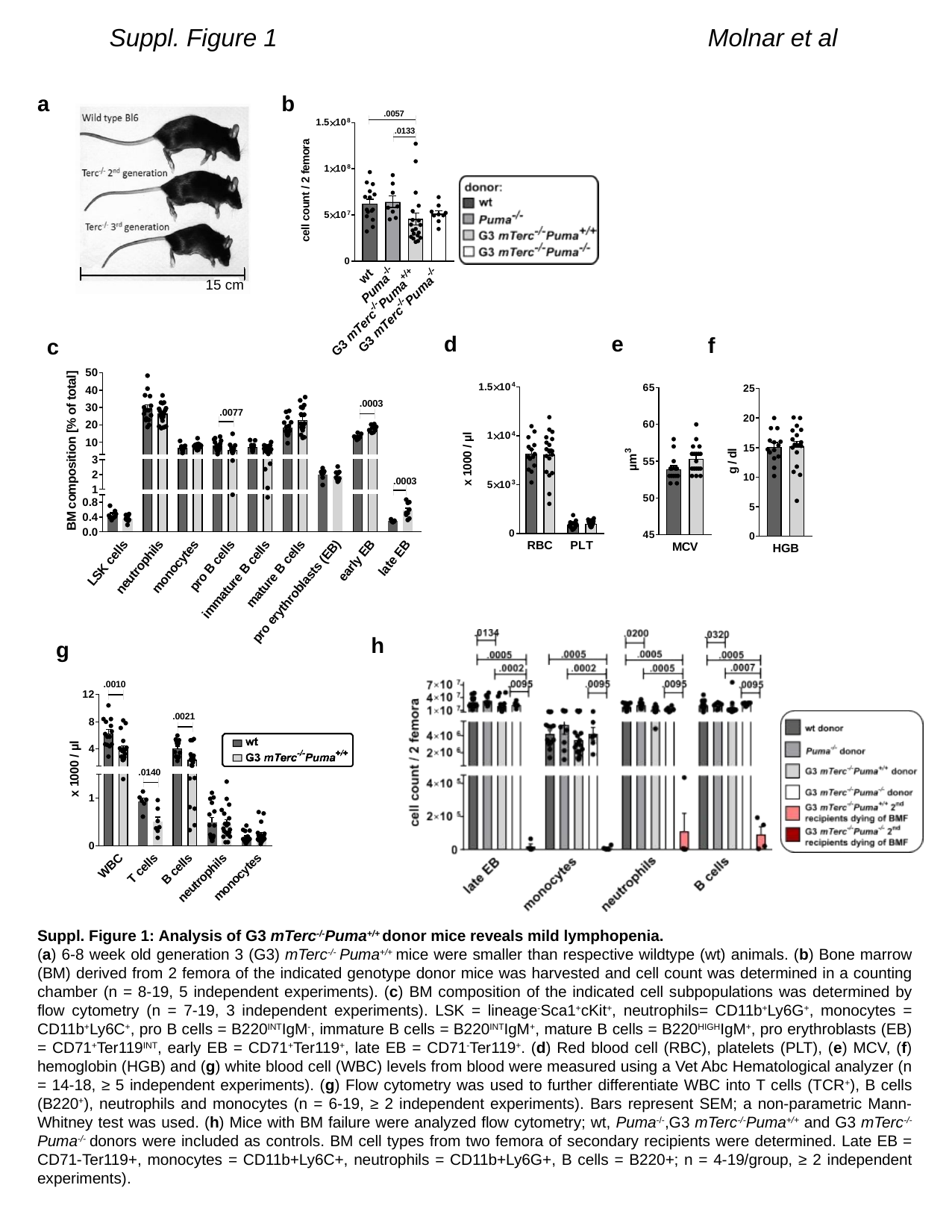

Suppl. Figure 1
Molnar et al
a
b
15 cm
d
e
f
c
h
g
Suppl. Figure 1: Analysis of G3 mTerc-/-Puma+/+ donor mice reveals mild lymphopenia.
(a) 6-8 week old generation 3 (G3) mTerc-/- Puma+/+ mice were smaller than respective wildtype (wt) animals. (b) Bone marrow (BM) derived from 2 femora of the indicated genotype donor mice was harvested and cell count was determined in a counting chamber (n = 8-19, 5 independent experiments). (c) BM composition of the indicated cell subpopulations was determined by flow cytometry (n = 7-19, 3 independent experiments). LSK = lineage-Sca1+cKit+, neutrophils= CD11b+Ly6G+, monocytes = CD11b+Ly6C+, pro B cells = B220INTIgM-, immature B cells = B220INTIgM+, mature B cells = B220HIGHIgM+, pro erythroblasts (EB) = CD71+Ter119INT, early EB = CD71+Ter119+, late EB = CD71-Ter119+. (d) Red blood cell (RBC), platelets (PLT), (e) MCV, (f) hemoglobin (HGB) and (g) white blood cell (WBC) levels from blood were measured using a Vet Abc Hematological analyzer (n = 14-18, ≥ 5 independent experiments). (g) Flow cytometry was used to further differentiate WBC into T cells (TCR+), B cells (B220+), neutrophils and monocytes (n = 6-19, ≥ 2 independent experiments). Bars represent SEM; a non-parametric Mann-Whitney test was used. (h) Mice with BM failure were analyzed flow cytometry; wt, Puma-/-,G3 mTerc-/-Puma+/+ and G3 mTerc-/-Puma-/- donors were included as controls. BM cell types from two femora of secondary recipients were determined. Late EB = CD71-Ter119+, monocytes = CD11b+Ly6C+, neutrophils = CD11b+Ly6G+, B cells = B220+; n = 4-19/group, ≥ 2 independent experiments).

## Slide 2
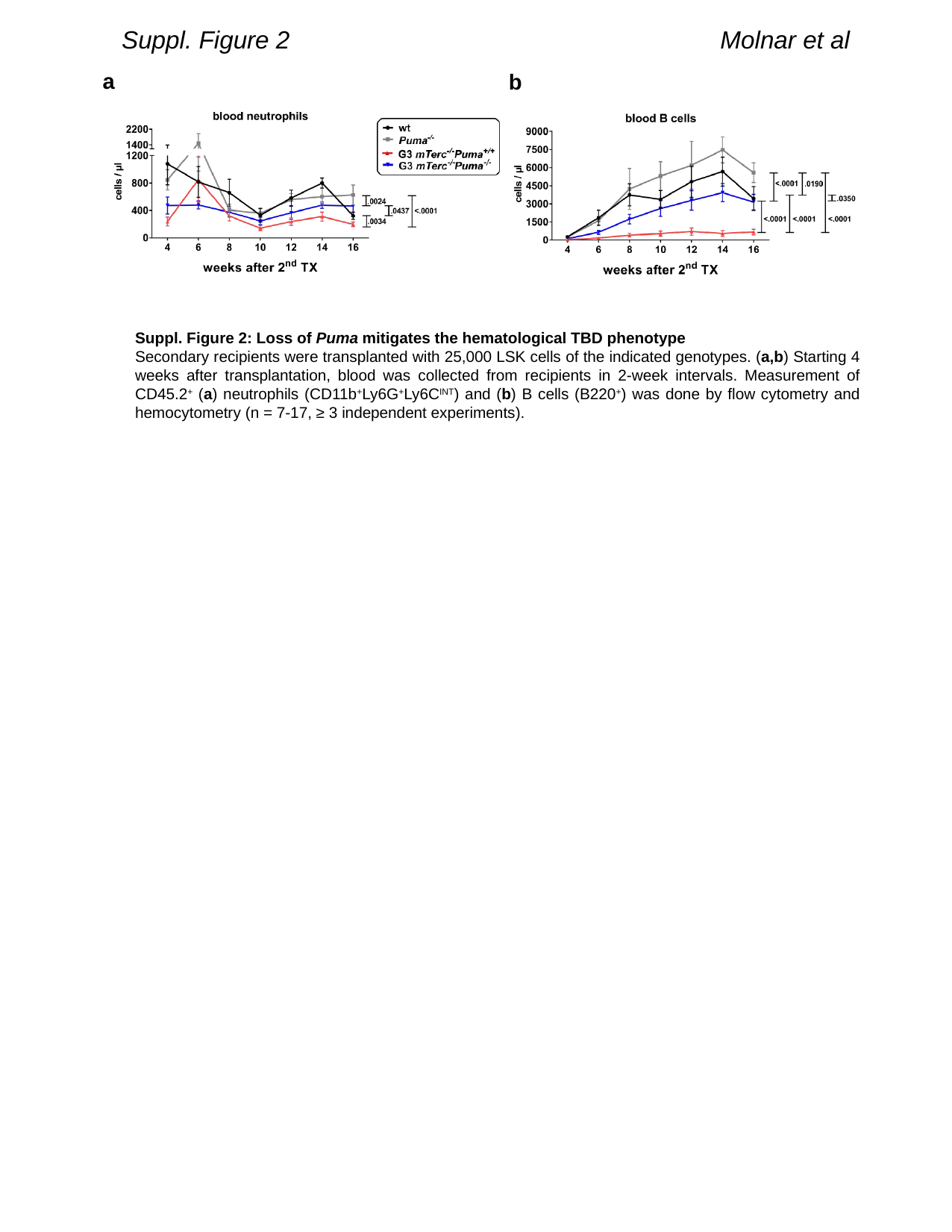

Suppl. Figure 2
Molnar et al
a
b
Suppl. Figure 2: Loss of Puma mitigates the hematological TBD phenotype
Secondary recipients were transplanted with 25,000 LSK cells of the indicated genotypes. (a,b) Starting 4 weeks after transplantation, blood was collected from recipients in 2-week intervals. Measurement of CD45.2+ (a) neutrophils (CD11b+Ly6G+Ly6CINT) and (b) B cells (B220+) was done by flow cytometry and hemocytometry (n = 7-17, ≥ 3 independent experiments).

## Slide 3
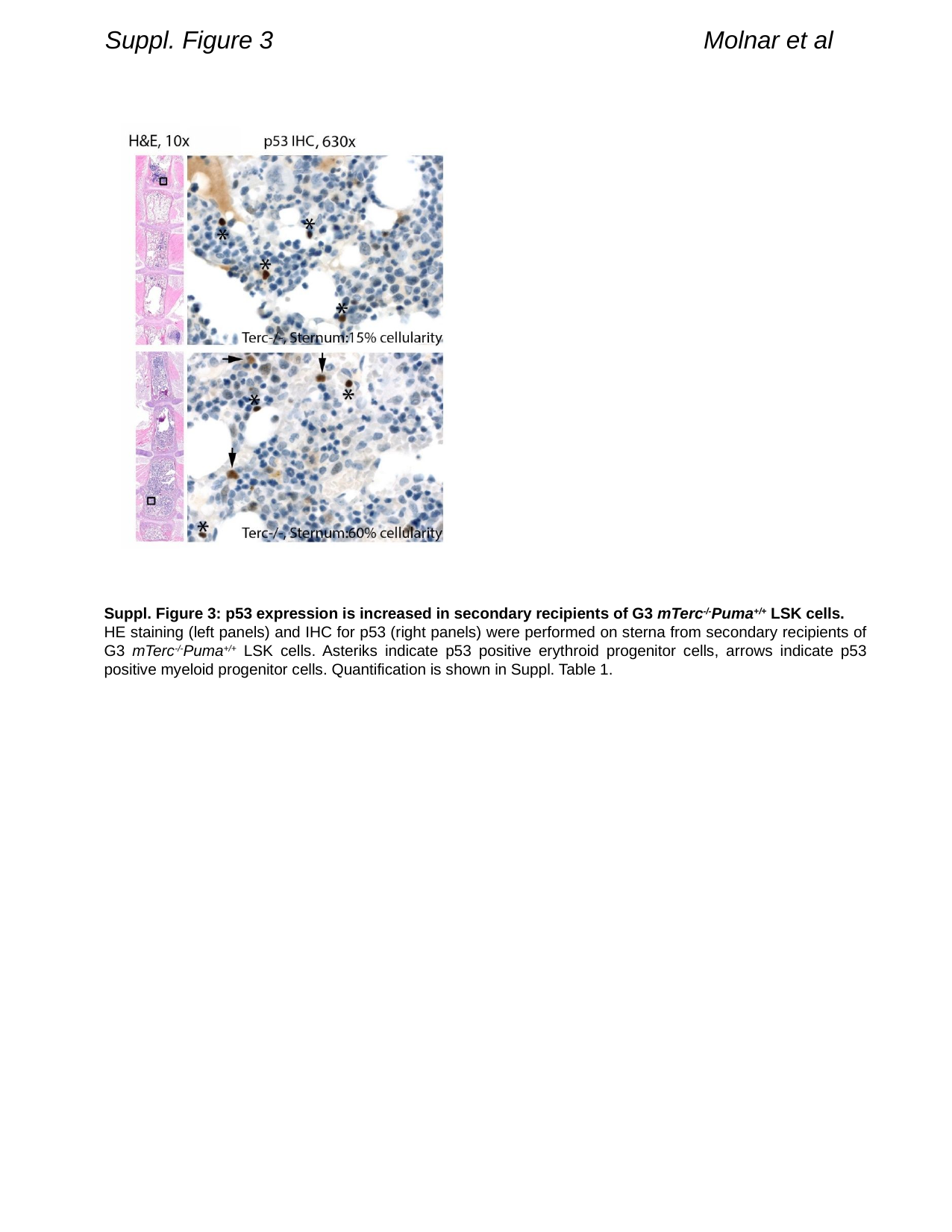

Suppl. Figure 3
Molnar et al
Suppl. Figure 3: p53 expression is increased in secondary recipients of G3 mTerc-/-Puma+/+ LSK cells.
HE staining (left panels) and IHC for p53 (right panels) were performed on sterna from secondary recipients of G3 mTerc-/-Puma+/+ LSK cells. Asteriks indicate p53 positive erythroid progenitor cells, arrows indicate p53 positive myeloid progenitor cells. Quantification is shown in Suppl. Table 1.

## Slide 4
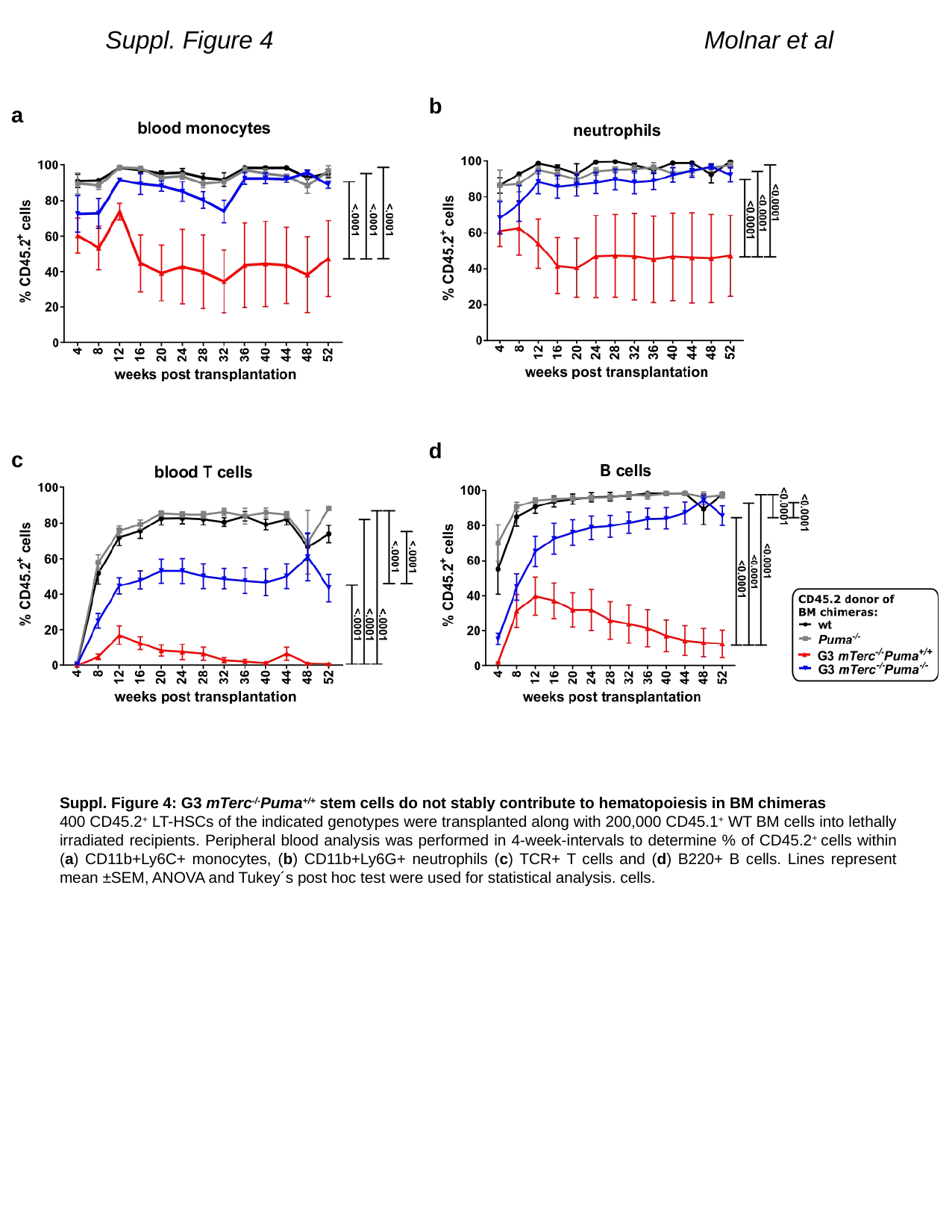

Suppl. Figure 4
Molnar et al
b
a
d
c
Suppl. Figure 4: G3 mTerc-/-Puma+/+ stem cells do not stably contribute to hematopoiesis in BM chimeras
400 CD45.2+ LT-HSCs of the indicated genotypes were transplanted along with 200,000 CD45.1+ WT BM cells into lethally irradiated recipients. Peripheral blood analysis was performed in 4-week-intervals to determine % of CD45.2+ cells within (a) CD11b+Ly6C+ monocytes, (b) CD11b+Ly6G+ neutrophils (c) TCR+ T cells and (d) B220+ B cells. Lines represent mean ±SEM, ANOVA and Tukey´s post hoc test were used for statistical analysis. cells.

## Slide 5
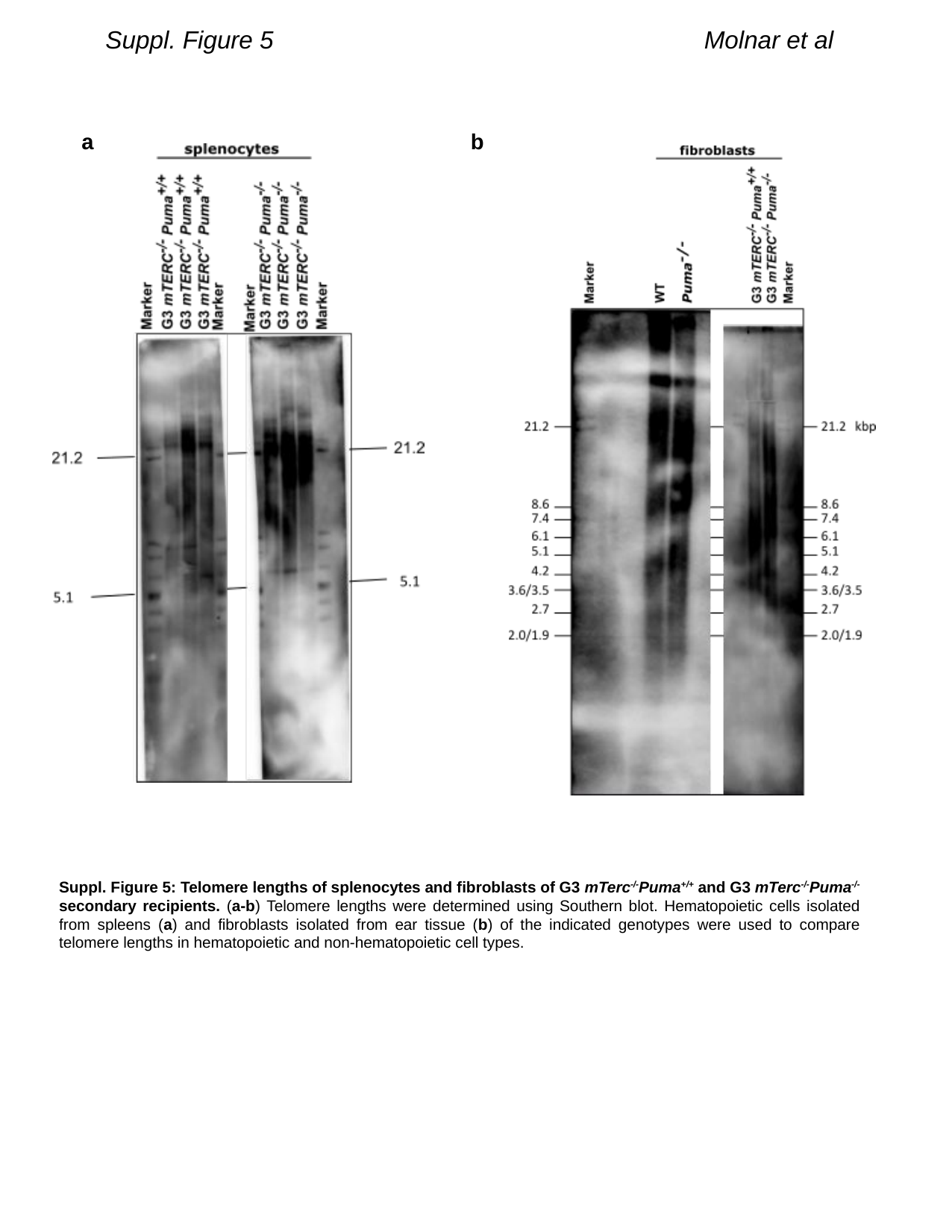

Suppl. Figure 5
Molnar et al
b
a
Suppl. Figure 5: Telomere lengths of splenocytes and fibroblasts of G3 mTerc-/-Puma+/+ and G3 mTerc-/-Puma-/- secondary recipients. (a-b) Telomere lengths were determined using Southern blot. Hematopoietic cells isolated from spleens (a) and fibroblasts isolated from ear tissue (b) of the indicated genotypes were used to compare telomere lengths in hematopoietic and non-hematopoietic cell types.

## Slide 6
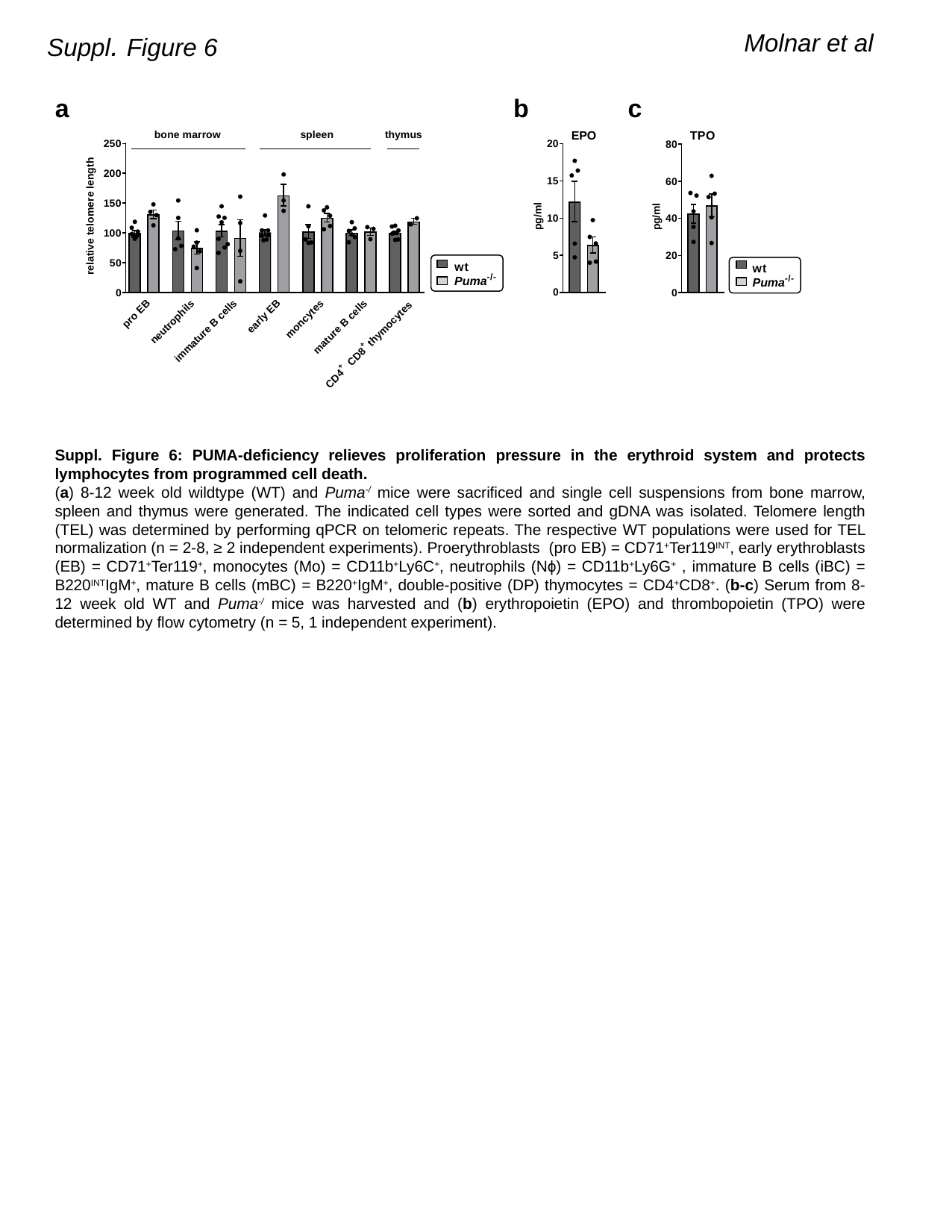

Suppl. Figure 6
Molnar et al
a
b
c
Suppl. Figure 6: PUMA-deficiency relieves proliferation pressure in the erythroid system and protects lymphocytes from programmed cell death.
(a) 8-12 week old wildtype (WT) and Puma-/ mice were sacrificed and single cell suspensions from bone marrow, spleen and thymus were generated. The indicated cell types were sorted and gDNA was isolated. Telomere length (TEL) was determined by performing qPCR on telomeric repeats. The respective WT populations were used for TEL normalization (n = 2-8, ≥ 2 independent experiments). Proerythroblasts (pro EB) = CD71+Ter119INT, early erythroblasts (EB) = CD71+Ter119+, monocytes (Mo) = CD11b+Ly6C+, neutrophils (Nϕ) = CD11b+Ly6G+ , immature B cells (iBC) = B220INTIgM+, mature B cells (mBC) = B220+IgM+, double-positive (DP) thymocytes = CD4+CD8+. (b-c) Serum from 8-12 week old WT and Puma-/ mice was harvested and (b) erythropoietin (EPO) and thrombopoietin (TPO) were determined by flow cytometry (n = 5, 1 independent experiment).

## Slide 7
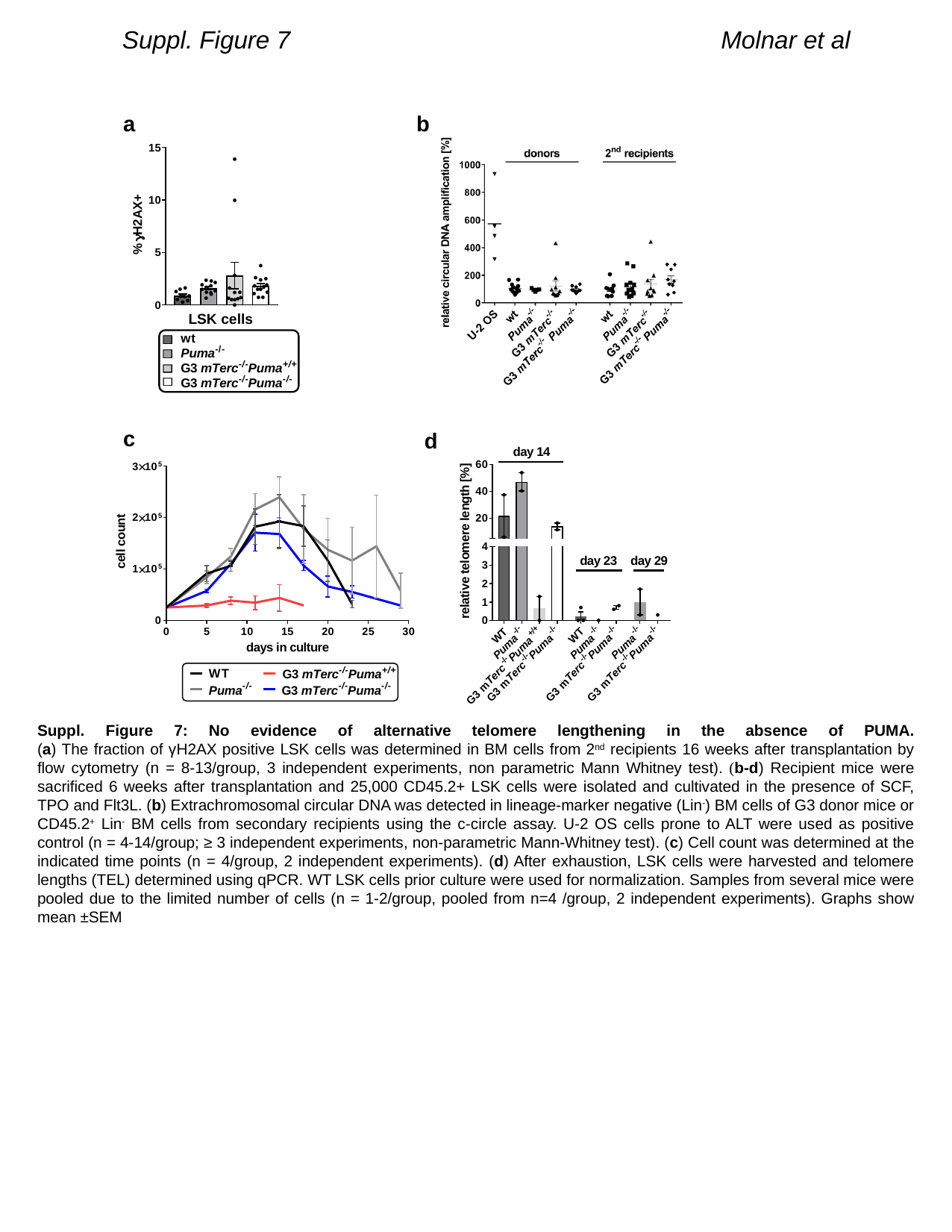

Suppl. Figure 7
Molnar et al
a
b
c
d
Suppl. Figure 7: No evidence of alternative telomere lengthening in the absence of PUMA.
(a) The fraction of γH2AX positive LSK cells was determined in BM cells from 2nd recipients 16 weeks after transplantation by flow cytometry (n = 8-13/group, 3 independent experiments, non parametric Mann Whitney test). (b-d) Recipient mice were sacrificed 6 weeks after transplantation and 25,000 CD45.2+ LSK cells were isolated and cultivated in the presence of SCF, TPO and Flt3L. (b) Extrachromosomal circular DNA was detected in lineage-marker negative (Lin-) BM cells of G3 donor mice or CD45.2+ Lin- BM cells from secondary recipients using the c-circle assay. U-2 OS cells prone to ALT were used as positive control (n = 4-14/group; ≥ 3 independent experiments, non-parametric Mann-Whitney test). (c) Cell count was determined at the indicated time points (n = 4/group, 2 independent experiments). (d) After exhaustion, LSK cells were harvested and telomere lengths (TEL) determined using qPCR. WT LSK cells prior culture were used for normalization. Samples from several mice were pooled due to the limited number of cells (n = 1-2/group, pooled from n=4 /group, 2 independent experiments). Graphs show mean ±SEM

## Slide 8
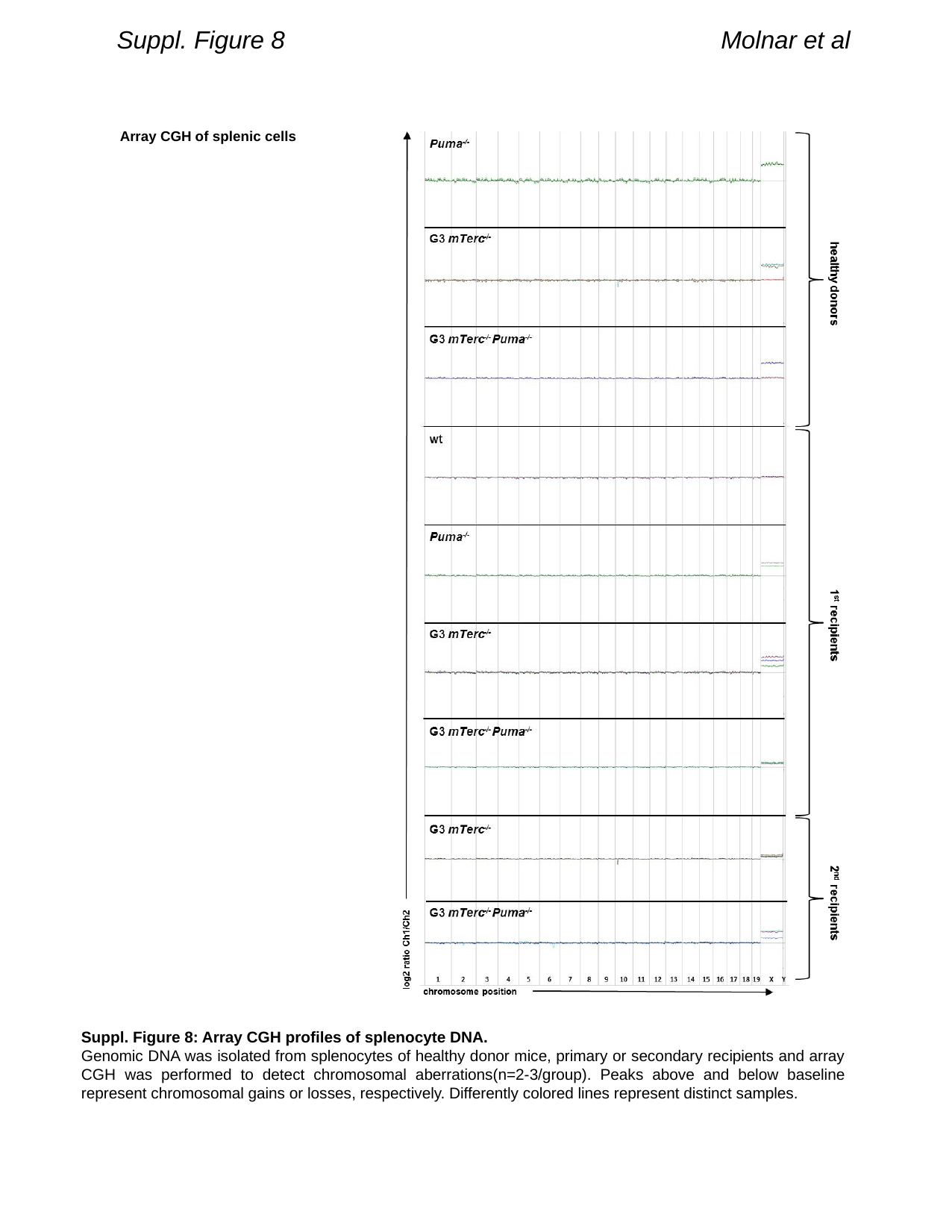

Suppl. Figure 8
Molnar et al
Array CGH of splenic cells
Suppl. Figure 8: Array CGH profiles of splenocyte DNA.
Genomic DNA was isolated from splenocytes of healthy donor mice, primary or secondary recipients and array CGH was performed to detect chromosomal aberrations(n=2-3/group). Peaks above and below baseline represent chromosomal gains or losses, respectively. Differently colored lines represent distinct samples.

## Slide 9
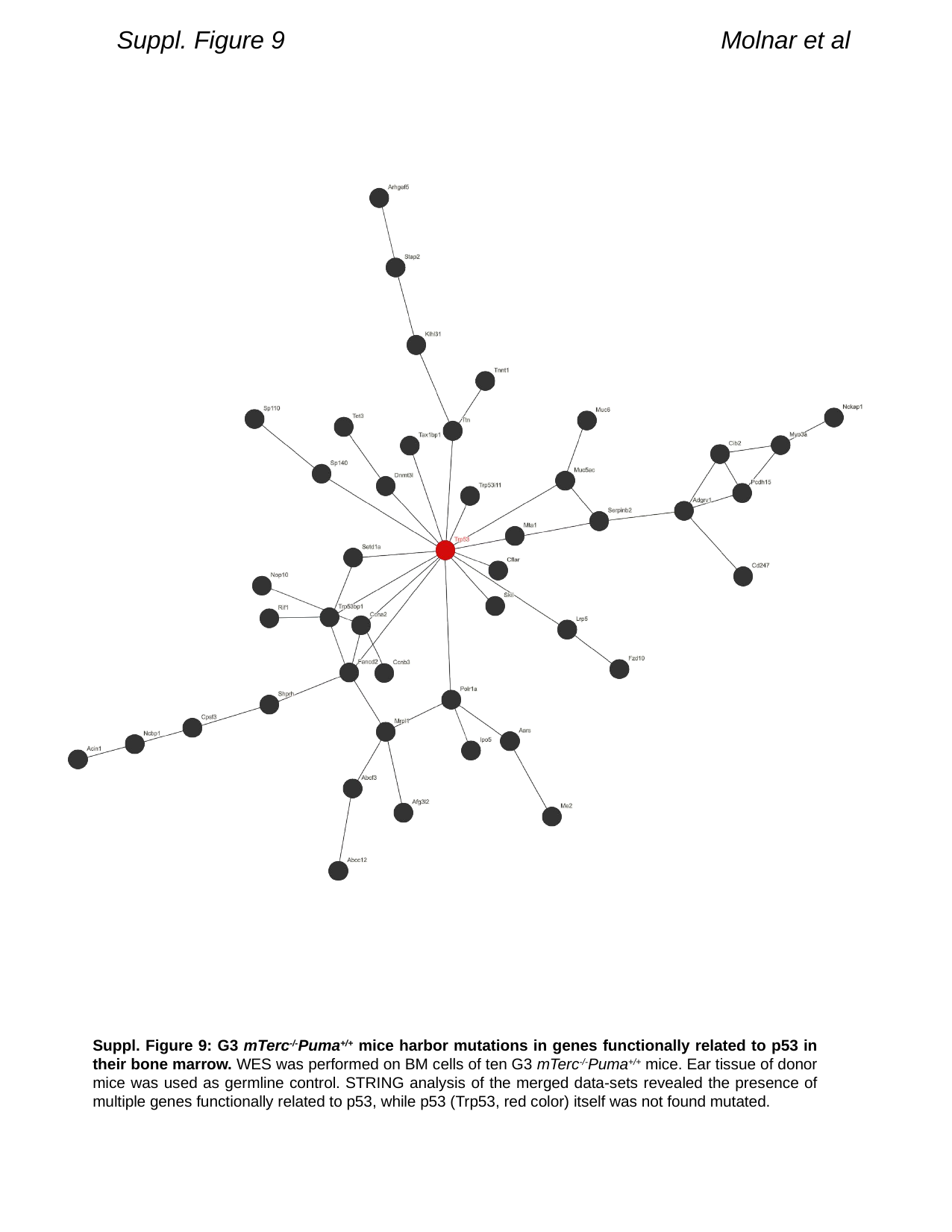

Suppl. Figure 9
Molnar et al
Suppl. Figure 9: G3 mTerc-/-Puma+/+ mice harbor mutations in genes functionally related to p53 in their bone marrow. WES was performed on BM cells of ten G3 mTerc-/-Puma+/+ mice. Ear tissue of donor mice was used as germline control. STRING analysis of the merged data-sets revealed the presence of multiple genes functionally related to p53, while p53 (Trp53, red color) itself was not found mutated.

## Slide 10
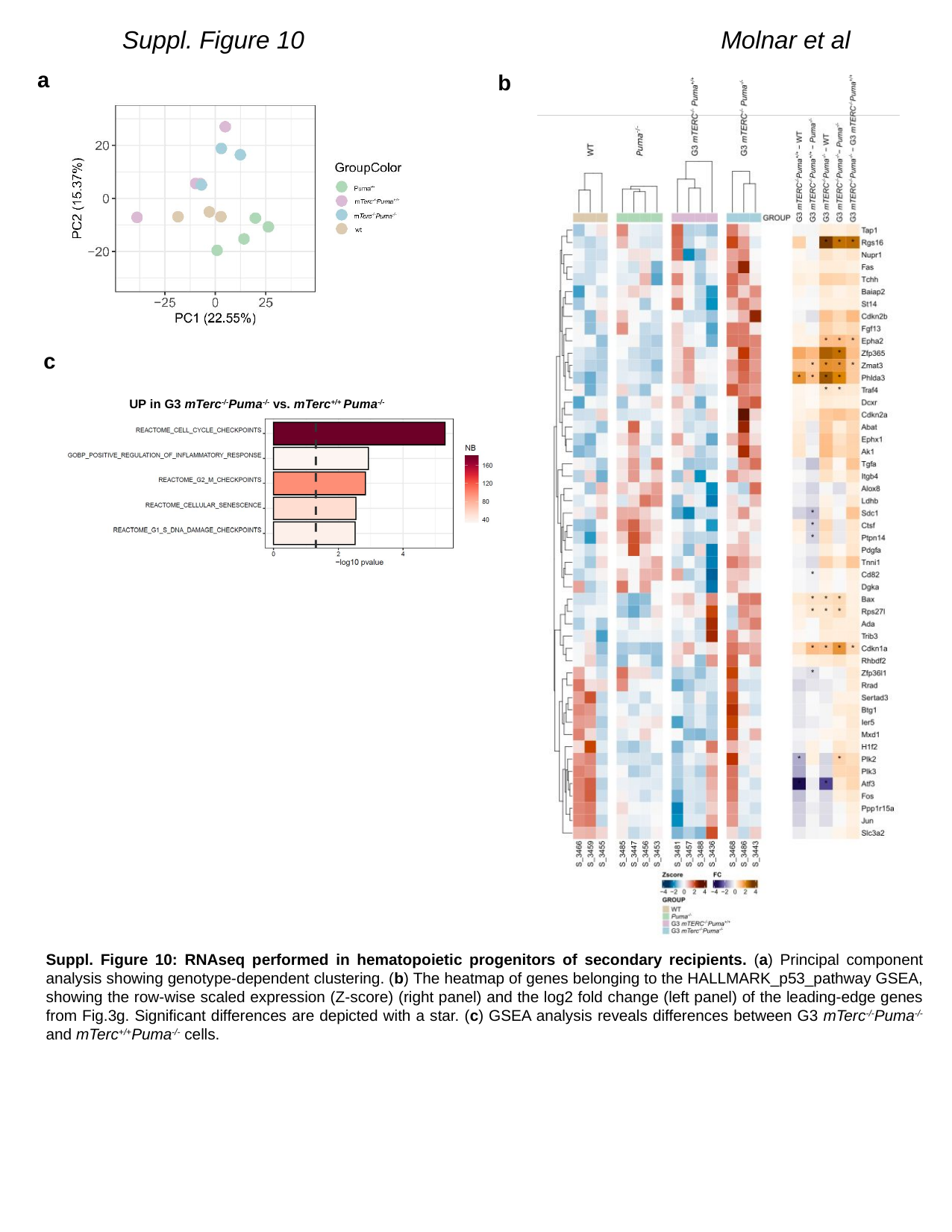

Suppl. Figure 10
Molnar et al
a
b
c
UP in G3 mTerc-/-Puma-/- vs. mTerc+/+ Puma-/-
Suppl. Figure 10: RNAseq performed in hematopoietic progenitors of secondary recipients. (a) Principal component analysis showing genotype-dependent clustering. (b) The heatmap of genes belonging to the HALLMARK_p53_pathway GSEA, showing the row-wise scaled expression (Z-score) (right panel) and the log2 fold change (left panel) of the leading-edge genes from Fig.3g. Significant differences are depicted with a star. (c) GSEA analysis reveals differences between G3 mTerc-/-Puma-/- and mTerc+/+Puma-/- cells.

## Slide 11
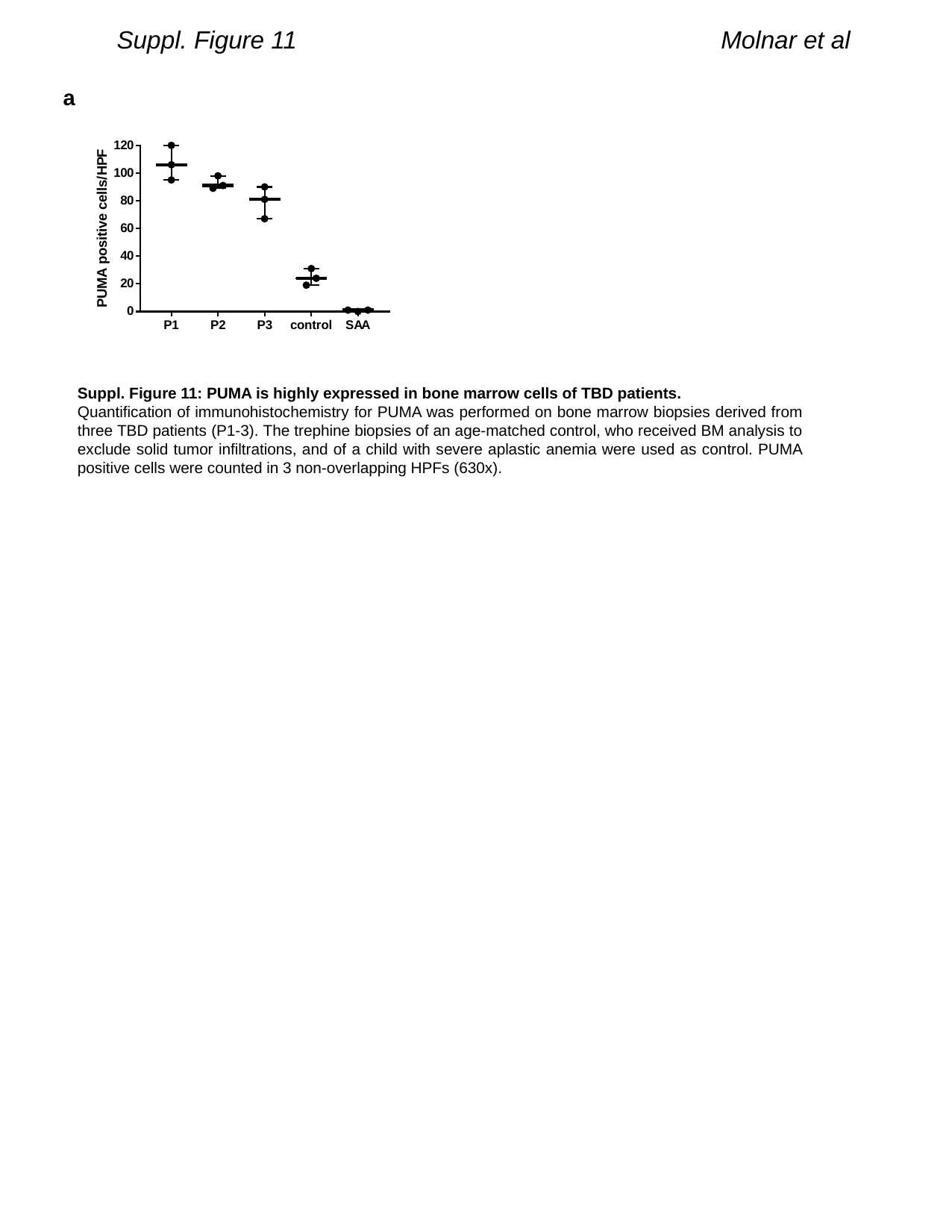

Suppl. Figure 11
Molnar et al
a
Suppl. Figure 11: PUMA is highly expressed in bone marrow cells of TBD patients.
Quantification of immunohistochemistry for PUMA was performed on bone marrow biopsies derived from three TBD patients (P1-3). The trephine biopsies of an age-matched control, who received BM analysis to exclude solid tumor infiltrations, and of a child with severe aplastic anemia were used as control. PUMA positive cells were counted in 3 non-overlapping HPFs (630x).

## Slide 12
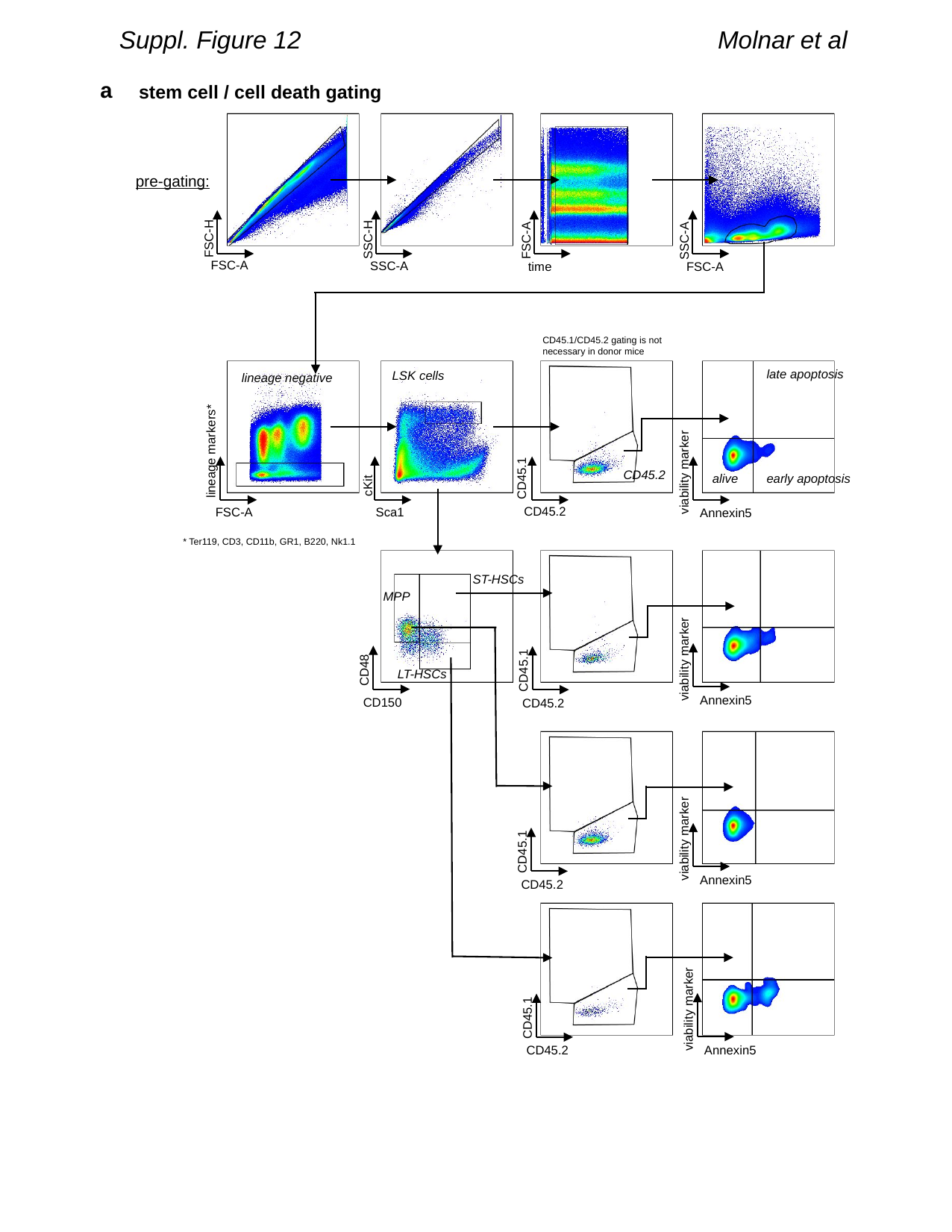

Suppl. Figure 12
Molnar et al
a
stem cell / cell death gating
pre-gating:
FSC-H
SSC-H
FSC-A
SSC-A
FSC-A
SSC-A
time
FSC-A
CD45.1/CD45.2 gating is not necessary in donor mice
late apoptosis
LSK cells
lineage negative
lineage markers*
viability marker
CD45.2
CD45.1
alive
early apoptosis
cKit
CD45.2
FSC-A
Sca1
Annexin5
* Ter119, CD3, CD11b, GR1, B220, Nk1.1
ST-HSCs
MPP
viability marker
CD48
CD45.1
LT-HSCs
Annexin5
CD150
CD45.2
viability marker
CD45.1
Annexin5
CD45.2
viability marker
CD45.1
Annexin5
CD45.2

## Slide 13
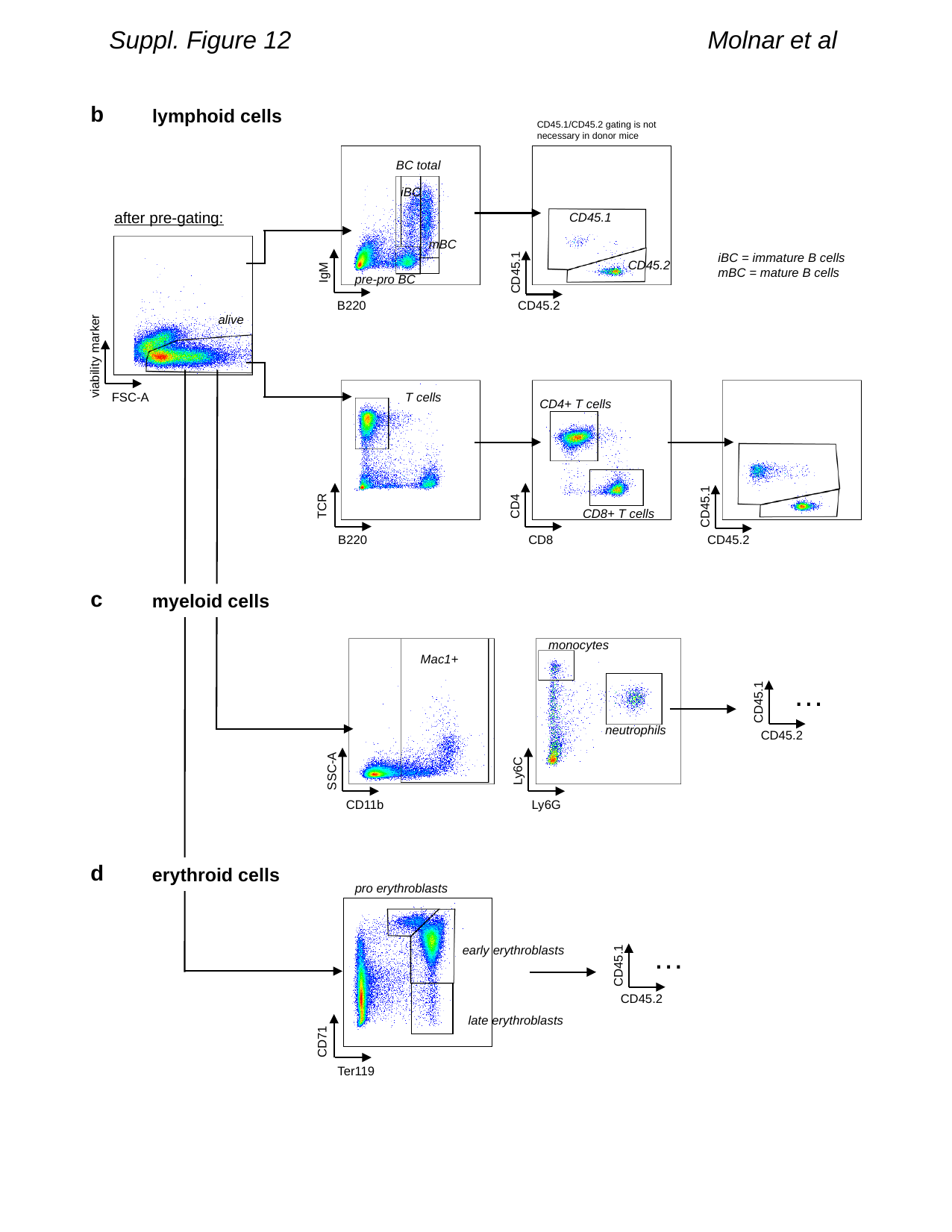

Suppl. Figure 12
Molnar et al
b
lymphoid cells
CD45.1/CD45.2 gating is not necessary in donor mice
BC total
iBC
after pre-gating:
CD45.1
mBC
CD45.1
CD45.2
iBC = immature B cells
mBC = mature B cells
IgM
B220
CD45.2
pre-pro BC
alive
viability marker
FSC-A
T cells
CD4+ T cells
CD45.1
CD45.2
TCR
B220
CD4
CD8
CD8+ T cells
c
myeloid cells
monocytes
Mac1+
CD45.1
CD45.2
…
neutrophils
SSC-A
CD11b
Ly6C
Ly6G
d
erythroid cells
pro erythroblasts
CD45.1
CD45.2
…
early erythroblasts
late erythroblasts
CD71
Ter119

## Slide 14
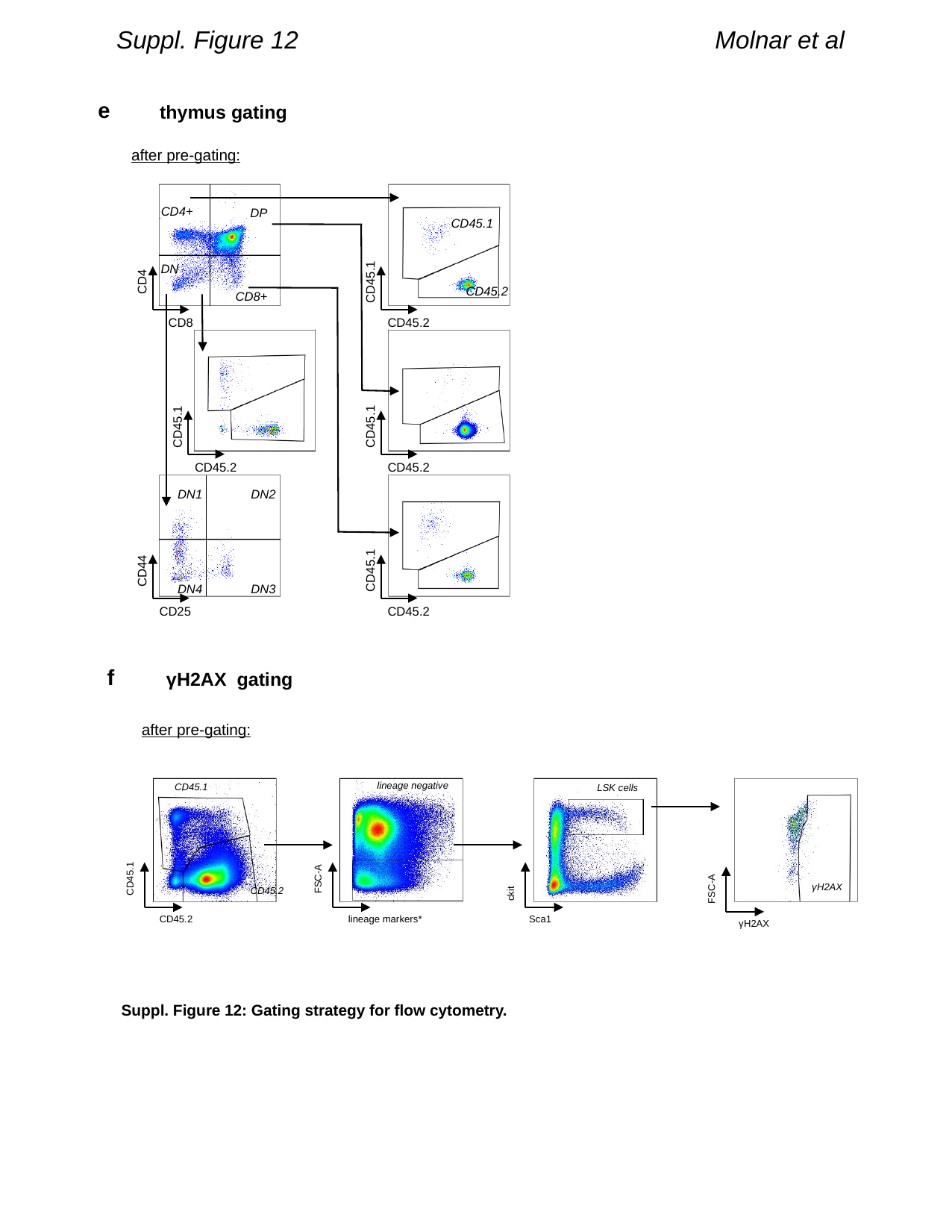

Suppl. Figure 12
Molnar et al
e
thymus gating
after pre-gating:
CD45.1
CD45.2
CD4
CD8
CD45.1
CD45.2
CD45.1
CD45.2
CD45.1
CD45.2
CD44
CD25
CD4+
DP
CD45.1
DN
CD45.2
CD8+
DN1
DN2
DN4
DN3
f
γH2AX gating
after pre-gating:
lineage negative
CD45.1
LSK cells
CD45.1
CD45.2
FSC-A
lineage markers*
FSC-A
γH2AX
ckit
Sca1
γH2AX
CD45.2
Suppl. Figure 12: Gating strategy for flow cytometry.

## Slide 15
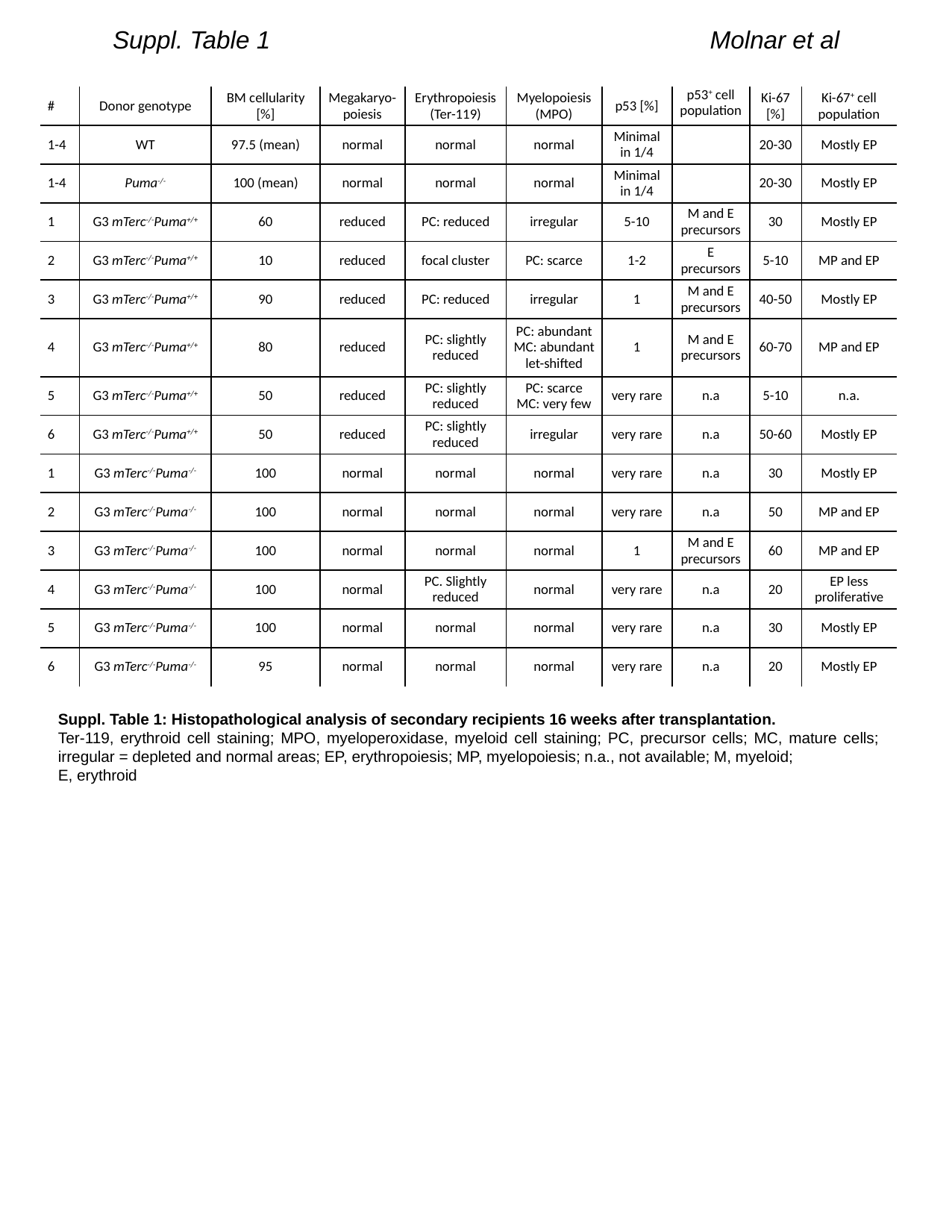

Suppl. Table 1
Molnar et al
Suppl. Table 1: Histopathological analysis of secondary recipients 16 weeks after transplantation.
Ter-119, erythroid cell staining; MPO, myeloperoxidase, myeloid cell staining; PC, precursor cells; MC, mature cells; irregular = depleted and normal areas; EP, erythropoiesis; MP, myelopoiesis; n.a., not available; M, myeloid;
E, erythroid
| # | Donor genotype | BM cellularity [%] | Megakaryo-poiesis | Erythropoiesis (Ter-119) | Myelopoiesis (MPO) | p53 [%] | p53+ cell population | Ki-67 [%] | Ki-67+ cell population |
| --- | --- | --- | --- | --- | --- | --- | --- | --- | --- |
| 1-4 | WT | 97.5 (mean) | normal | normal | normal | Minimal in 1/4 | | 20-30 | Mostly EP |
| 1-4 | Puma-/- | 100 (mean) | normal | normal | normal | Minimal in 1/4 | | 20-30 | Mostly EP |
| 1 | G3 mTerc-/-Puma+/+ | 60 | reduced | PC: reduced | irregular | 5-10 | M and E precursors | 30 | Mostly EP |
| 2 | G3 mTerc-/-Puma+/+ | 10 | reduced | focal cluster | PC: scarce | 1-2 | E precursors | 5-10 | MP and EP |
| 3 | G3 mTerc-/-Puma+/+ | 90 | reduced | PC: reduced | irregular | 1 | M and E precursors | 40-50 | Mostly EP |
| 4 | G3 mTerc-/-Puma+/+ | 80 | reduced | PC: slightly reduced | PC: abundant MC: abundant let-shifted | 1 | M and E precursors | 60-70 | MP and EP |
| 5 | G3 mTerc-/-Puma+/+ | 50 | reduced | PC: slightly reduced | PC: scarce MC: very few | very rare | n.a | 5-10 | n.a. |
| 6 | G3 mTerc-/-Puma+/+ | 50 | reduced | PC: slightly reduced | irregular | very rare | n.a | 50-60 | Mostly EP |
| 1 | G3 mTerc-/-Puma-/- | 100 | normal | normal | normal | very rare | n.a | 30 | Mostly EP |
| 2 | G3 mTerc-/-Puma-/- | 100 | normal | normal | normal | very rare | n.a | 50 | MP and EP |
| 3 | G3 mTerc-/-Puma-/- | 100 | normal | normal | normal | 1 | M and E precursors | 60 | MP and EP |
| 4 | G3 mTerc-/-Puma-/- | 100 | normal | PC. Slightly reduced | normal | very rare | n.a | 20 | EP less proliferative |
| 5 | G3 mTerc-/-Puma-/- | 100 | normal | normal | normal | very rare | n.a | 30 | Mostly EP |
| 6 | G3 mTerc-/-Puma-/- | 95 | normal | normal | normal | very rare | n.a | 20 | Mostly EP |

## Slide 16
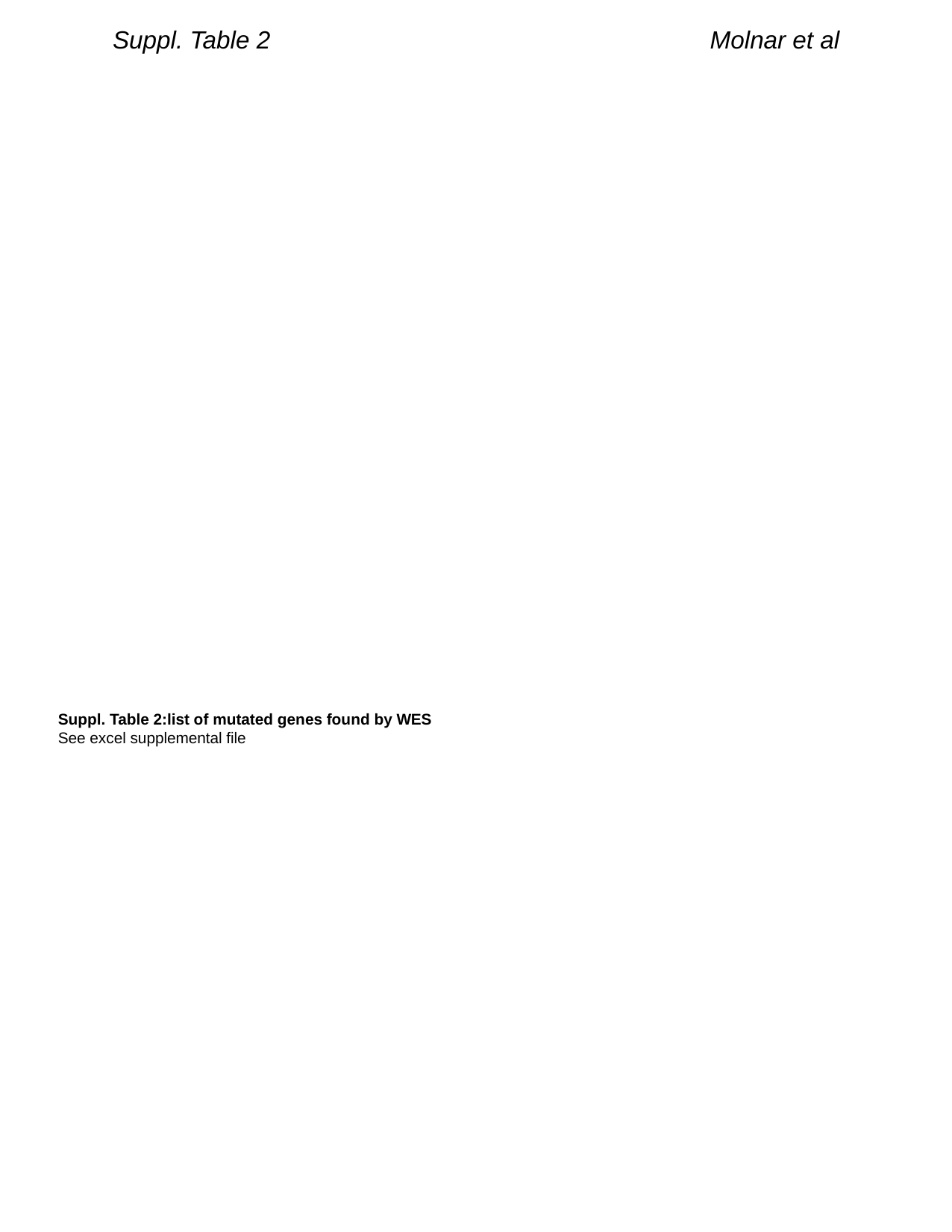

Suppl. Table 2
Molnar et al
Suppl. Table 2:list of mutated genes found by WES
See excel supplemental file

## Slide 17
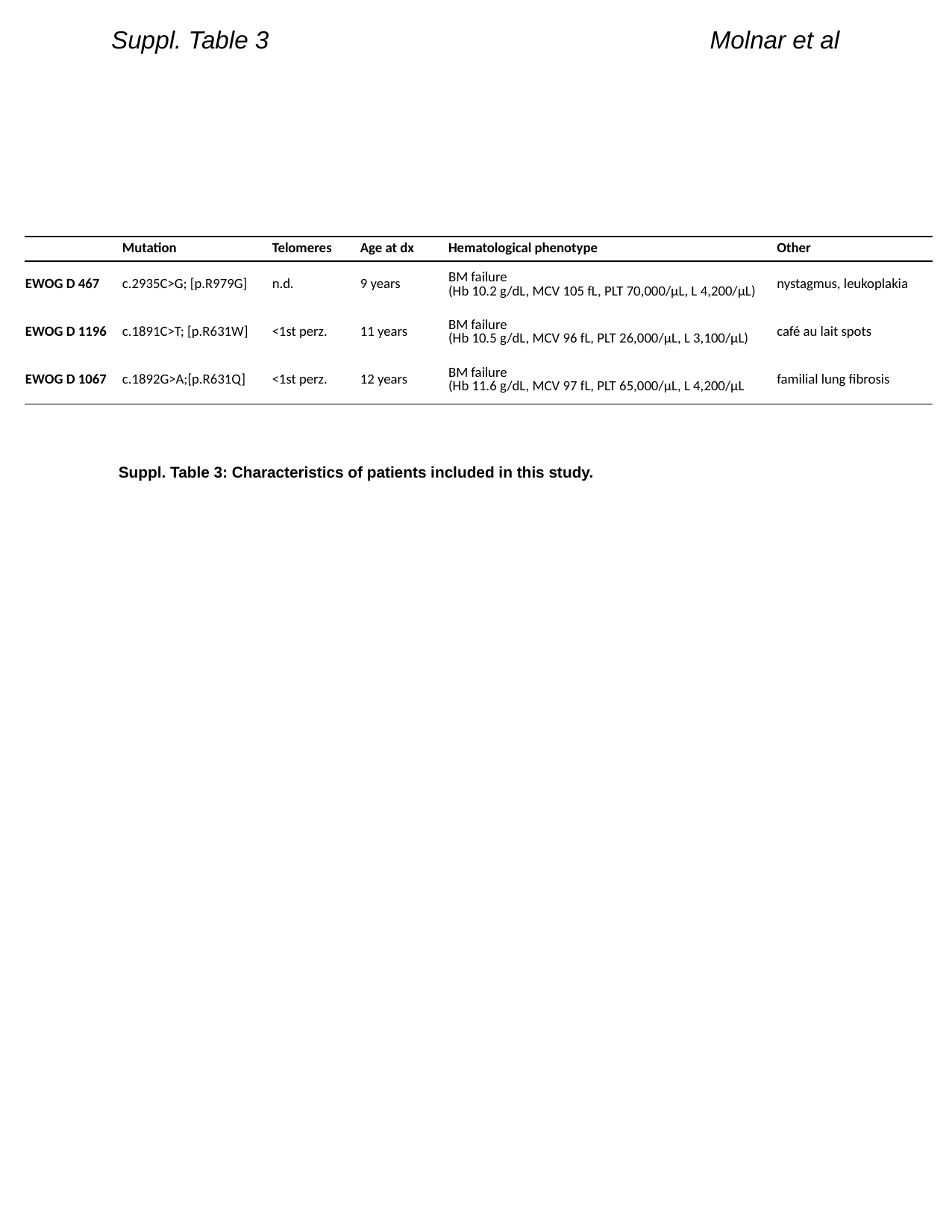

Suppl. Table 3
Molnar et al
| | Mutation | Telomeres | Age at dx | Hematological phenotype | Other |
| --- | --- | --- | --- | --- | --- |
| EWOG D 467 | c.2935C>G; [p.R979G] | n.d. | 9 years | BM failure (Hb 10.2 g/dL, MCV 105 fL, PLT 70,000/µL, L 4,200/µL) | nystagmus, leukoplakia |
| EWOG D 1196 | c.1891C>T; [p.R631W] | <1st perz. | 11 years | BM failure (Hb 10.5 g/dL, MCV 96 fL, PLT 26,000/µL, L 3,100/µL) | café au lait spots |
| EWOG D 1067 | c.1892G>A;[p.R631Q] | <1st perz. | 12 years | BM failure (Hb 11.6 g/dL, MCV 97 fL, PLT 65,000/µL, L 4,200/µL | familial lung fibrosis |
Suppl. Table 3: Characteristics of patients included in this study.

## Slide 18
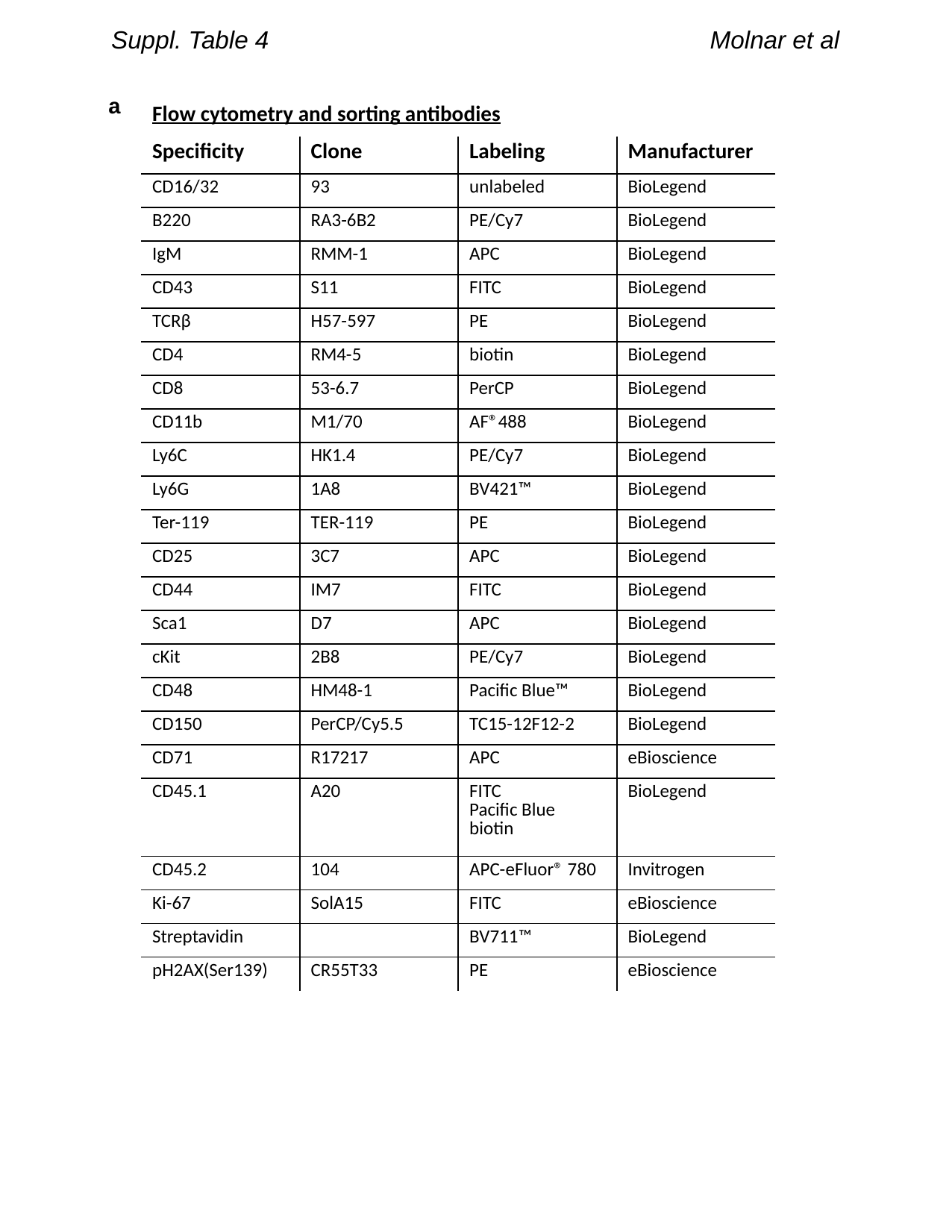

Suppl. Table 4
Molnar et al
a
| Flow cytometry and sorting antibodies | | | |
| --- | --- | --- | --- |
| Specificity | Clone | Labeling | Manufacturer |
| CD16/32 | 93 | unlabeled | BioLegend |
| B220 | RA3-6B2 | PE/Cy7 | BioLegend |
| IgM | RMM-1 | APC | BioLegend |
| CD43 | S11 | FITC | BioLegend |
| TCRβ | H57-597 | PE | BioLegend |
| CD4 | RM4-5 | biotin | BioLegend |
| CD8 | 53-6.7 | PerCP | BioLegend |
| CD11b | M1/70 | AF®488 | BioLegend |
| Ly6C | HK1.4 | PE/Cy7 | BioLegend |
| Ly6G | 1A8 | BV421™ | BioLegend |
| Ter-119 | TER-119 | PE | BioLegend |
| CD25 | 3C7 | APC | BioLegend |
| CD44 | IM7 | FITC | BioLegend |
| Sca1 | D7 | APC | BioLegend |
| cKit | 2B8 | PE/Cy7 | BioLegend |
| CD48 | HM48-1 | Pacific Blue™ | BioLegend |
| CD150 | PerCP/Cy5.5 | TC15-12F12-2 | BioLegend |
| CD71 | R17217 | APC | eBioscience |
| CD45.1 | A20 | FITC Pacific Blue biotin | BioLegend |
| CD45.2 | 104 | APC-eFluor® 780 | Invitrogen |
| Ki-67 | SolA15 | FITC | eBioscience |
| Streptavidin | | BV711™ | BioLegend |
| pH2AX(Ser139) | CR55T33 | PE | eBioscience |

## Slide 19
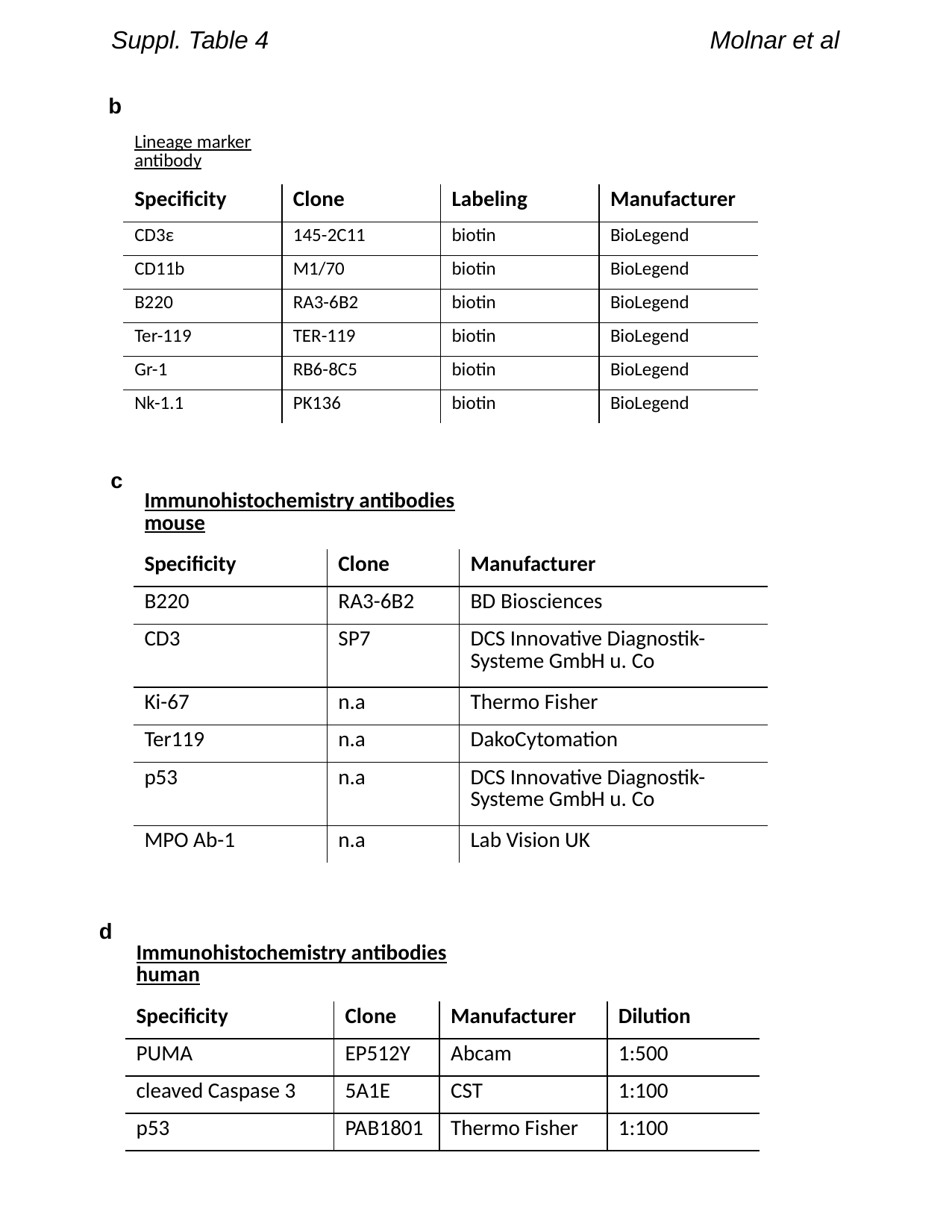

Suppl. Table 4
Molnar et al
b
| Lineage marker antibody | | | |
| --- | --- | --- | --- |
| Specificity | Clone | Labeling | Manufacturer |
| CD3ε | 145-2C11 | biotin | BioLegend |
| CD11b | M1/70 | biotin | BioLegend |
| B220 | RA3-6B2 | biotin | BioLegend |
| Ter-119 | TER-119 | biotin | BioLegend |
| Gr-1 | RB6-8C5 | biotin | BioLegend |
| Nk-1.1 | PK136 | biotin | BioLegend |
c
| Immunohistochemistry antibodies mouse | | |
| --- | --- | --- |
| Specificity | Clone | Manufacturer |
| B220 | RA3-6B2 | BD Biosciences |
| CD3 | SP7 | DCS Innovative Diagnostik-Systeme GmbH u. Co |
| Ki-67 | n.a | Thermo Fisher |
| Ter119 | n.a | DakoCytomation |
| p53 | n.a | DCS Innovative Diagnostik-Systeme GmbH u. Co |
| MPO Ab-1 | n.a | Lab Vision UK |
d
| Immunohistochemistry antibodies human | | | |
| --- | --- | --- | --- |
| Specificity | Clone | Manufacturer | Dilution |
| PUMA | EP512Y | Abcam | 1:500 |
| cleaved Caspase 3 | 5A1E | CST | 1:100 |
| p53 | PAB1801 | Thermo Fisher | 1:100 |

## Slide 20
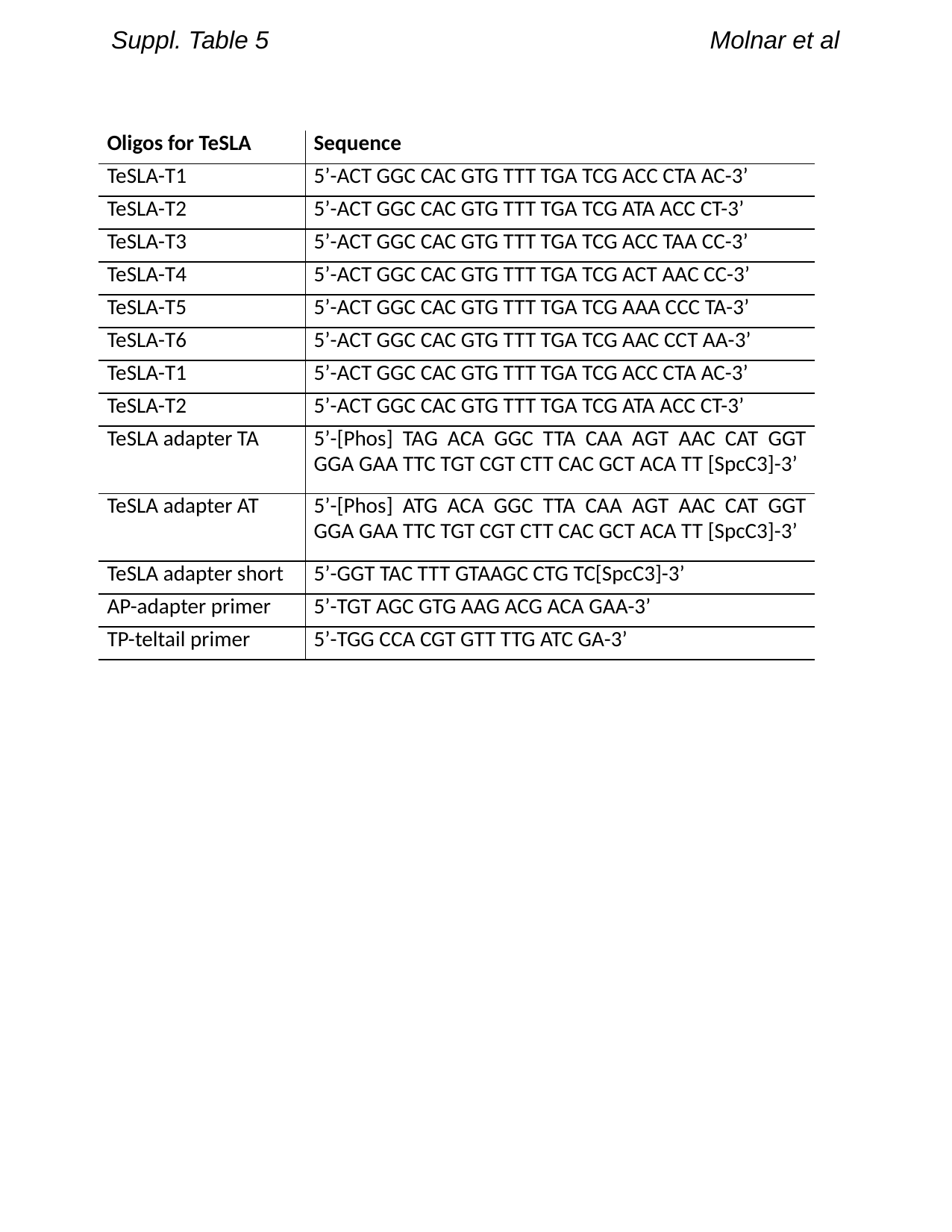

Suppl. Table 5
Molnar et al
| Oligos for TeSLA | Sequence |
| --- | --- |
| TeSLA-T1 | 5’-ACT GGC CAC GTG TTT TGA TCG ACC CTA AC-3’ |
| TeSLA-T2 | 5’-ACT GGC CAC GTG TTT TGA TCG ATA ACC CT-3’ |
| TeSLA-T3 | 5’-ACT GGC CAC GTG TTT TGA TCG ACC TAA CC-3’ |
| TeSLA-T4 | 5’-ACT GGC CAC GTG TTT TGA TCG ACT AAC CC-3’ |
| TeSLA-T5 | 5’-ACT GGC CAC GTG TTT TGA TCG AAA CCC TA-3’ |
| TeSLA-T6 | 5’-ACT GGC CAC GTG TTT TGA TCG AAC CCT AA-3’ |
| TeSLA-T1 | 5’-ACT GGC CAC GTG TTT TGA TCG ACC CTA AC-3’ |
| TeSLA-T2 | 5’-ACT GGC CAC GTG TTT TGA TCG ATA ACC CT-3’ |
| TeSLA adapter TA | 5’-[Phos] TAG ACA GGC TTA CAA AGT AAC CAT GGT GGA GAA TTC TGT CGT CTT CAC GCT ACA TT [SpcC3]-3’ |
| TeSLA adapter AT | 5’-[Phos] ATG ACA GGC TTA CAA AGT AAC CAT GGT GGA GAA TTC TGT CGT CTT CAC GCT ACA TT [SpcC3]-3’ |
| TeSLA adapter short | 5’-GGT TAC TTT GTAAGC CTG TC[SpcC3]-3’ |
| AP-adapter primer | 5’-TGT AGC GTG AAG ACG ACA GAA-3’ |
| TP-teltail primer | 5’-TGG CCA CGT GTT TTG ATC GA-3’ |

## Slide 21
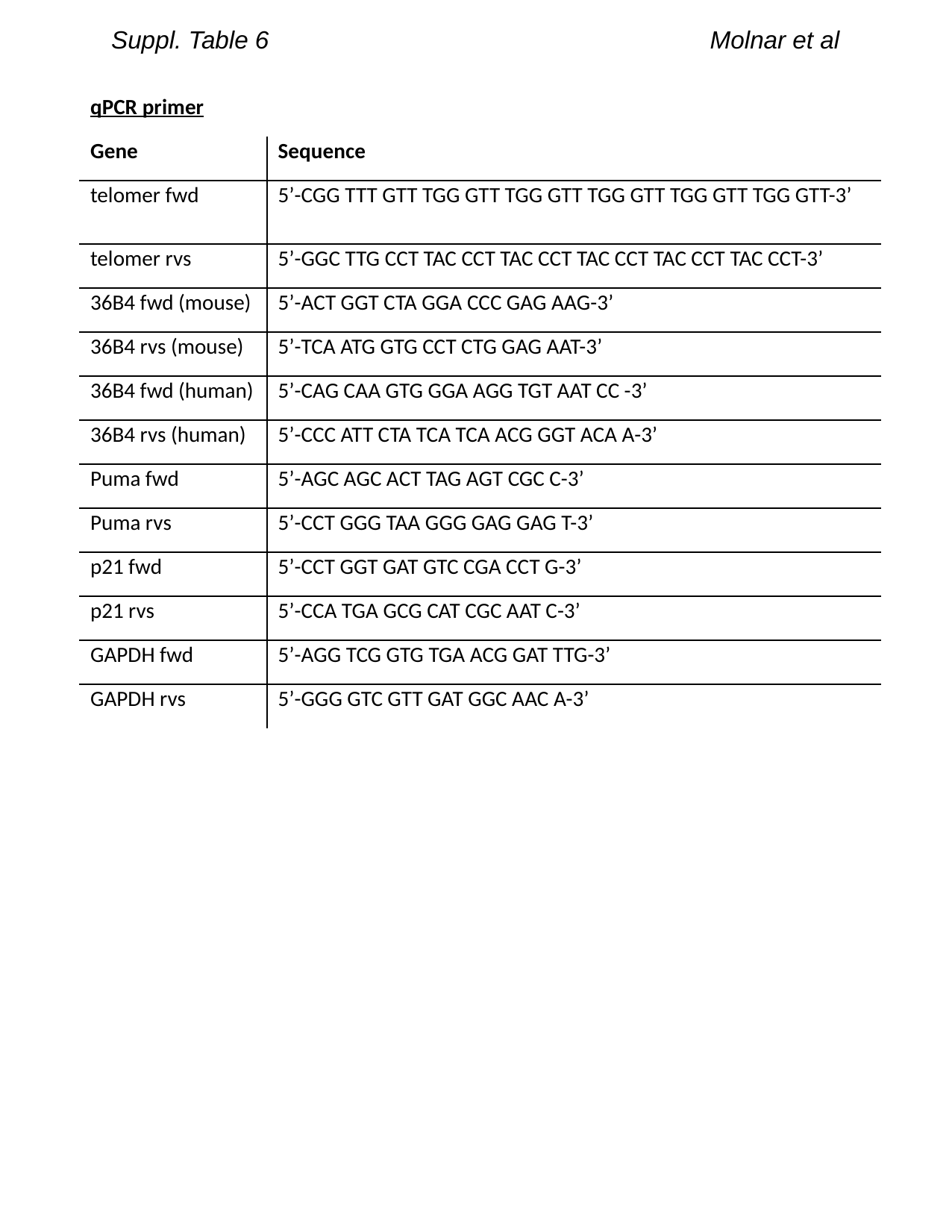

Suppl. Table 6
Molnar et al
| qPCR primer | |
| --- | --- |
| Gene | Sequence |
| telomer fwd | 5’-CGG TTT GTT TGG GTT TGG GTT TGG GTT TGG GTT TGG GTT-3’ |
| telomer rvs | 5’-GGC TTG CCT TAC CCT TAC CCT TAC CCT TAC CCT TAC CCT-3’ |
| 36B4 fwd (mouse) | 5’-ACT GGT CTA GGA CCC GAG AAG-3’ |
| 36B4 rvs (mouse) | 5’-TCA ATG GTG CCT CTG GAG AAT-3’ |
| 36B4 fwd (human) | 5’-CAG CAA GTG GGA AGG TGT AAT CC -3’ |
| 36B4 rvs (human) | 5’-CCC ATT CTA TCA TCA ACG GGT ACA A-3’ |
| Puma fwd | 5’-AGC AGC ACT TAG AGT CGC C-3’ |
| Puma rvs | 5’-CCT GGG TAA GGG GAG GAG T-3’ |
| p21 fwd | 5’-CCT GGT GAT GTC CGA CCT G-3’ |
| p21 rvs | 5’-CCA TGA GCG CAT CGC AAT C-3’ |
| GAPDH fwd | 5’-AGG TCG GTG TGA ACG GAT TTG-3’ |
| GAPDH rvs | 5’-GGG GTC GTT GAT GGC AAC A-3’ |
