# Supplementary material for: PUMA-induced apoptosis drives bone marrow failure and genomic instability in telomerase-deficient mice
Source: Cell Death Differ. 2025 Aug 19;33(1):38–50. doi: 10.1038/s41418-025-01557-w (PMC12811261; doi:10.1038/s41418-025-01557-w)
Supplement: Supplementary file 2 — Supplemental table 2 [file 41418_2025_1557_MOESM2_ESM.pdf]

Mouse numb	VAF,cov	Chr	Start	End	Ref	Alt	Func.refGene	Gene.refGene	GeneDetail.re	ExonicFunc.re	AAChange.ref	Entrez.mouse	Symbol.huma	Entrez.humar	Chr.human	Gene.Start.hu	Gene.End.human
T1	VAF:6.02%; cov:133	chr1	127469063	127469063	T	-	exonic	Slc35f5		frameshift del	Slc35f5:NM_0	74150	SLC35F5	80255	2	114462588	114514400
T1	VAF:7.32%; cov:164	chr5	72636963	72636963	G	T	exonic	Atp10d		unknown	UNKNOWN	231287	ATP10D	57205	4	47487305	47595503
T2	VAF:5.57%; cov:1167	chr5	14914792	14914792	C	A	exonic	Gm9758		nonsynonymo	Gm9758:NM_	381714					
T2	VAF:5.63%; cov:284	chr1	85250732	85250732	A	G	exonic	C130026I21Rik		nonsynonymo	C130026I21Ri	620078					
T2	VAF:8.94%; cov:179	chr14	52033554	52033554	A	C	exonic	Vmn2r88		nonsynonymo	Vmn2r88:NM_	669149					
T2	VAF:87.5%; cov:8	chr6	52210543	52210543	-	G	exonic;splicin	Hoxa13;Hoxa1	NM_008264:e	unknown	UNKNOWN	15398	HOXA13	3209	7	27233122	27239725
T3	VAF:6.03%; cov:116	chr4	131942837	131942837	T	-	exonic	Phactr4		frameshift del	Phactr4:NM_(	100169	PHACTR4	65979	1	28696114	28826881
T4	VAF:35.85%; cov:53	chr1	85097247	85097247	C	T	exonic	A530032D15Rik		nonsynonymo	A530032D15R	381287					
T4	VAF:5%; cov:140	chr5	96693349	96693349	C	A	exonic	Mrpl1		nonsynonymo	Mrpl1:NM_05	94061	MRPL1	65008	4	78783674	78873944
T4	VAF:5.02%; cov:239	chr12	32394760	32394760	C	A	exonic	Gpr22		nonsynonymo	Gpr22:NM_17	73010	GPR22	2845	7	107110463	107116098
T4	VAF:5.03%; cov:159	chr7	148993239	148993239	C	A	exonic	Muc5ac		nonsynonymo	Muc5ac:NM_(	17833	MUC5AC	4586	11	1151580	1222364
T4	VAF:5.03%; cov:199	chr16	35933151	35933151	C	A	exonic	Dtx3l		nonsynonymo	Dtx3l:NM_001	209200	DTX3L	151636	3	122283085	122294050
T4	VAF:5.13%; cov:156	chr2	80363600	80363600	C	A	exonic	Nckap1		nonsynonymo	Nckap1:NM_(	50884	NCKAP1	10787	2	183773843	183903586
T4	VAF:5.22%; cov:134	chr12	119253832	119253832	C	A	exonic	Dnah11		nonsynonymo	Dnah11:NM_(	13411	DNAH11	8701	7	21582833	21941457
T4	VAF:5.23%; cov:153	chr18	36910902	36910902	C	A	exonic	Ik		nonsynonymo	Ik:NM_01187	24010	IK	3550	5	140026643	140042064
T4	VAF:5.23%; cov:172	chr1	87996397	87996397	C	A	exonic	Htr2b		nonsynonymo	Htr2b:NM_00	15559	HTR2B	3357	2	231972944	231989832
T4	VAF:5.26%; cov:133	chr9	107503377	107503377	C	A	exonic	Sema3b		nonsynonymo	Sema3b:NM_(	20347	SEMA3B	7869	3	50304990	50314977
T4	VAF:5.43%; cov:129	chr13	63117119	63117119	C	A	exonic	2010111I01Rik		nonsynonymo	2010111I01Rik:NM_001289924:exon2:c.C593A:p.A198D,2010111I01Rik:NM_001289926:exon2:c.C593A:p.A198D						
T4	VAF:5.47%; cov:128	chr14	61372694	61372694	C	A	exonic	Spata13		nonsynonymo	Spata13:NM_(	219140	SPATA13	221178	13	24553949	24609166
T4	VAF:5.56%; cov:126	chr8	40134557	40134557	C	A	exonic	Tusc3		nonsynonymo	Tusc3:NM_00	80286	TUSC3	7991	8	15274724	15624158
T4	VAF:5.56%; cov:162	chr7	4463879	4463879	C	A	exonic	Tnnt1		nonsynonymo	Tnnt1:NM_00	21955	TNNT1	7138	19	55644162	55660722
T4	VAF:5.65%; cov:124	chr6	52683258	52683258	C	A	exonic	Tax1bp1		nonsynonymo	Tax1bp1:NM_	52440	TAX1BP1	8887	7	27778950	27884183
T4	VAF:5.67%; cov:141	chr11	94356280	94356280	C	A	exonic	Epn3		nonsynonymo	Epn3:NM_027	71889	EPN3	55040	17	48609904	48621111
T4	VAF:5.79%; cov:121	chr9	50703516	50703516	C	A	exonic	Sik2		nonsynonymo	Sik2:NM_1787	235344	SIK2	23235	11	111473115	111601577
T4	VAF:5.98%; cov:117	chr2	153704195	153704195	C	A	exonic	Bpifb2		nonsynonymo	Bpifb2:NM_0.	66557	BPIFB2	80341	20	31595406	31611515
T4	VAF:6.14%; cov:114	chr3	104456907	104456907	C	A	exonic	Slc16a1		nonsynonymo	Slc16a1:NM_(	20501	SLC16A1	6566	1	113454469	113499635
T4	VAF:6.25%; cov:112	chr1	20965053	20965053	C	A	exonic	Efhc1		nonsynonymo	Efhc1:NM_027	71877	EFHC1	114327	6	52285106	52387892
T4	VAF:6.54%; cov:107	chr14	121340146	121340146	C	A	exonic	Ipo5		nonsynonymo	Ipo5:NM_001	70572	IPO5	3843	13	98605912	98676551
T4	VAF:6.67%; cov:105	chr3	36469824	36469824	C	A	exonic	Ccna2		nonsynonymo	Ccna2:NM_00	12428	CCNA2	890	4	122737599	122745087
T4	VAF:6.86%; cov:102	chr3	137819778	137819778	C	A	exonic	Trmt10a		nonsynonymo	Trmt10a:NM_	108943	TRMT10A	93587	4	100467866	100485189
T4	VAF:7.45%; cov:94	chr14	24973880	24973880	C	A	exonic	Dlg5		nonsynonymo	Dlg5:NM_027	71228	DLG5	9231	10	79550549	79686378
T4	VAF:7.55%; cov:106	chr9	77503029	77503029	C	A	exonic	Klhl31		nonsynonymo	Klhl31:NM_17	244923	KLHL31	401265	6	53512699	53530506
T4	VAF:7.62%; cov:105	chr10	38752823	38752823	C	A	exonic	Lama4		nonsynonymo	Lama4:NM_0.	16775	LAMA4	3910	6	112429963	112576141
T4	VAF:7.87%; cov:89	chr2	25149996	25149996	C	A	exonic	Grin1		nonsynonymo	Grin1:NM_008	14810	GRIN1	2902	9	140032842	140063207
T5	VAF:10.45%; cov:67	chrX	3907981	3907981	G	A	exonic	Btbd35f21;Btbd35f23;Btbd3		nonsynonymo	Btbd35f28:NM	100862329					
T5	VAF:16.29%; cov:350	chr1	85257437	85257437	C	T	exonic	C130026I21Rik		nonsynonymo	C130026I21Ri	620078					
T5	VAF:26.57%; cov:399	chr5	26327637	26327637	G	T	exonic	Gm1979		nonsynonymo	Gm1979:NM_	100038949					
T5	VAF:5.13%; cov:390	chr8	19937337	19937337	T	A	exonic	Gm15319;Gm21119		nonsynonymo	Gm15319:NM	100040599					
T5	VAF:5.28%; cov:379	chr8	19937336	19937336	G	A	exonic	Gm15319;Gm21119		stopgain	Gm15319:NM	100040599					
T5	VAF:6.1%; cov:885	chr1	87490926	87490926	T	G	exonic	Sp110		nonsynonymo	Sp110:NM_03	109032	SP110	3431	2	231032009	231090444
T5	VAF:7.91%; cov:215	chr4	57722863	57722863	A	G	exonic	Palm2		nonsynonymo	Palm2:NM_172868:exon6:c.A935G:p.D312G						
T5	VAF:82.17%; cov:1335	chr10	57693973	57693973	C	T	exonic	Dux		nonsynonymo	Dux:NM_0010	664783					
T5	VAF:82.35%; cov:17	chr6	52210398	52210398	-	GC	exonic;splicin	Hoxa13;Hoxa1	NM_008264:e	unknown	UNKNOWN	15398	HOXA13	3209	7	27233122	27239725
T6	VAF:10.14%; cov:69	chr9	106273090	106273090	C	A	exonic	Dusp7		nonsynonymo	Dusp7:NM_15	235584	DUSP7	1849	3	52082935	52090566
T6	VAF:14.29%; cov:112	chr8	13055400	13055400	G	A	exonic	F10		nonsynonymo	F10:NM_0012	14058	F10	2159	13	113777128	113803843
T6	VAF:20.25%; cov:79	chrX	59543557	59543557	C	T	exonic	4933402E13Rik		nonsynonymo	4933402E13Rik:NM_001199996:exon4:c.C7T:p.R3C,4933402E13Rik:NM_001199997:exon4:c.C7T:p.R3C						
T6	VAF:35.29%; cov:17	chr4	80557529	80557529	C	A	exonic	Lurap1l		nonsynonymo	Lurap1l:NM_(	52829	LURAP1L	286343	9	12775020	12822130
T6	VAF:5%; cov:140	chr4	126803394	126803394	C	A	exonic	Tmem35b		nonsynonymo	Tmem35b:NM	100039968	TMEM35B	100506144	1	35447136	35450954
T6	VAF:5%; cov:160	chr17	54109033	54109033	C	A	exonic	Sult1c1		nonsynonymo	Sult1c1:NM_C	20888					
T6	VAF:5.06%; cov:158	chr9	64349379	64349379	C	A	exonic	Megf11		nonsynonymo	Megf11:NM_(	214058	MEGF11	84465	HSCHR15_1_(	66187417	66558222
T6	VAF:5.07%; cov:138	chr5	105368183	105368183	C	A	exonic	Abcg3		nonsynonymo	Abcg3:NM_03	27405					
T6	VAF:5.1%; cov:157	chr13	104840790	104840790	C	A	exonic	Nln		nonsynonymo	Nln:NM_0294	75805	NLN	57486	5	65018023	65167553
T6	VAF:5.11%; cov:137	chr15	75935994	75935994	C	A	exonic	Eppk1		nonsynonymo	Eppk1:NM_14	223650	EPPK1	83481	HG104_HG97	144940157	144955423
T6	VAF:5.11%; cov:137	chr2	90749973	90749973	C	A	exonic	Kbtbd4		nonsynonymo	Kbtbd4:NM_0	67136	KBTBD4	55709	11	47593749	47600567
T6	VAF:5.11%; cov:137	chr6	83336045	83336045	C	A	exonic	Tet3		nonsynonymo	Tet3:NM_183	194388	TET3	200424	2	74229840	74335303
T6	VAF:5.13%; cov:156	chr4	46174980	46174980	C	A	exonic	Ncbp1		nonsynonymo	Ncbp1:NM_0(	433702	NCBP1	4686	9	100395908	100436030
T6	VAF:5.15%; cov:136	chr12	114369968	114369968	C	A	exonic	Mta1		nonsynonymo	Mta1:NM_054	116870	MTA1	9112	14	105886159	105937066
T6	VAF:5.19%; cov:135	chr11	113655154	113655154	C	A	exonic	Sdk2		stopgain	Sdk2:NM_172	237979	SDK2	54549	17	71330523	71640228
T6	VAF:5.19%; cov:135	chr2	155735149	155735149	C	A	exonic	Uqcc1		nonsynonymo	Uqcc1:NM_00	56046	UQCC1	55245	20	33890369	33999944
T6	VAF:5.19%; cov:135	chr5	124583461	124583461	C	A	exonic	Pitpnm2		stopgain	Pitpnm2:NM_	19679	PITPNM2	57605	12	123468027	123634562
T6	VAF:5.19%; cov:154	chr7	149003779	149003779	C	A	exonic	Muc5ac		nonsynonymo	Muc5ac:NM_(	17833	MUC5AC	4586	11	1151580	1222364
T6	VAF:5.23%; cov:153	chr18	37892879	37892879	C	A	exonic	Pcdhgb5		nonsynonymo	Pcdhgb5:NM_	93702	PCDHGB5	56101	5	140767452	140892546
T6	VAF:5.26%; cov:133	chr12	56858978	56858978	C	A	exonic	Ralgapa1		nonsynonymo	Ralgapa1:NM_	56784	RALGAPA1	253959	14	36007558	36278510
T6	VAF:5.3%; cov:151	chr13	54742840	54742840	C	A	exonic	Faf2		nonsynonymo	Faf2:NM_1783	76577	FAF2	23197	5	175874629	175937075
T6	VAF:5.34%; cov:131	chr9	7861041	7861041	C	A	exonic	Birc3		nonsynonymo	Birc3:NM_007	11796	BIRC3	330	11	102188215	102210134
T6	VAF:5.37%; cov:149	chr7	110654756	110654756	C	A	exonic	Olfr610		stopgain	Olfr610:NM_1	259085					
T6	VAF:5.38%; cov:130	chr14	35490505	35490505	C	A	exonic	Wapl		nonsynonymo	Wapl:NM_001	218914	WAPL	23063	10	88195013	88281572
T6	VAF:5.38%; cov:130	chr2	24947318	24947318	C	A	exonic	Noxa1		nonsynonymo	Noxa1:NM_0(	241275	NOXA1	10811	9	140317802	140328858
T6	VAF:5.38%; cov:186	chr7	35756686	35756686	C	A	exonic	Pepd		nonsynonymo	Pepd:NM_008	18624	PEPD	5184	19	33877856	34012700
T6	VAF:5.39%; cov:167	chr13	81643099	81643099	C	A	exonic	Adgrv1		nonsynonymo	Adgrv1:NM_0	110789	ADGRV1	84059	5	89825161	90460038
T6	VAF:5.41%; cov:185	chr6	113534911	113534911	C	A	exonic	Fancd2		nonsynonymo	Fancd2:NM_0	211651	FANCD2	2177	3	10068098	10143614
T6	VAF:5.43%; cov:129	chr6	43222434	43222434	C	A	exonic	Arhgef5		nonsynonymo	Arhgef5:NM_	54324	ARHGEF5	7984	7	144052381	144077725
T6	VAF:5.43%; cov:129	chr7	29671423	29671423	C	A	exonic	Capn12		nonsynonymo	Capn12:NM_(	60594	CAPN12	147968	19	39220827	39260544
T6	VAF:5.43%; cov:129	chr9	119526921	119526921	C	A	exonic	Scn10a		nonsynonymo	Scn10a:NM_0	20264	SCN10A	6336	3	38738293	38835501
T6	VAF:5.44%; cov:147	chr4	123150268	123150268	C	A	exonic	Macf1		stopgain	Macf1:NM_0(	11426	MACF1	23499	1	39546988	39952849
T6	VAF:5.47%; cov:128	chr15	101611399	101611399	C	A	exonic	Krt72		nonsynonymo	Krt72:NM_21	105866	KRT72	140807	12	52979373	52995322
T6	VAF:5.47%; cov:128	chr18	73954125	73954125	C	A	exonic	Me2		stopgain	Me2:NM_145	107029	ME2	4200	18	48405419	48474691
T6	VAF:5.51%; cov:127	chr1	109421221	109421221	C	A	exonic	Serpinb2		nonsynonymo	Serpinb2:NM_	18788	SERPINB2	5055	18	61538926	61571124

T6	VAF:5.51%; cov:127	chr1	58809572	58809572	C	A	exonic	Cflar	nonsynonymo	Cflar:NM_001	12633	CFLAR	8837	2	201980827	202041410
T6	VAF:5.56%; cov:126	chr10	118649570	118649570	C	A	exonic	Cand1	nonsynonymo	Cand1:NM_02	71902	CAND1	55832	12	67663061	67713731
T6	VAF:5.56%; cov:126	chr10	77520054	77520054	C	A	exonic	Dnmt3l	nonsynonymo	Dnmt3l:NM_0	54427	DNMT3L	29947	21	45666222	45682099
T6	VAF:5.56%; cov:126	chr6	40529451	40529451	C	A	exonic	Clec5a	stopgain	Clec5a:NM_02	23845	CLEC5A	23601	HG7_PATCH	141627157	141646807
T6	VAF:5.56%; cov:144	chr19	3612501	3612501	C	A	exonic	Lrp5	nonsynonymo	Lrp5:NM_008	16973	LRP5	4041	11	68080077	68216743
T6	VAF:5.56%; cov:144	chr2	23255738	23255738	C	A	exonic	Nxph2	nonsynonymo	Nxph2:NM_00	18232	NXPH2	11249	2	139428342	139537918
T6	VAF:5.6%; cov:125	chr8	113566208	113566208	C	A	exonic	Aars	nonsynonymo	Aars:NM_146	234734					
T6	VAF:5.62%; cov:178	chr18	15611392	15611392	C	A	exonic	Chst9	nonsynonymo	Chst9:NM_19	71367	CHST9	83539	18	24495595	24765281
T6	VAF:5.63%; cov:142	chr2	14882312	14882312	C	A	exonic	Cacnb2	nonsynonymo	Cacnb2:NM_0	12296	CACNB2	783	10	18429606	18830798
T6	VAF:5.65%; cov:124	chr2	142199967	142199967	C	A	exonic	Macrod2	nonsynonymo	Macrod2:NM_	72899	MACROD2	140733	20	13976015	16033842
T6	VAF:5.65%; cov:124	chr2	51966242	51966242	C	A	exonic	Rif1	nonsynonymo	Rif1:NM_001	51869	RIF1	55183	2	152266397	152364527
T6	VAF:5.65%; cov:124	chr8	126908401	126908401	C	A	exonic	Pgbd5	nonsynonymo	Pgbd5:NM_17	209966	PGBD5	79605	1	230457392	230561475
T6	VAF:5.68%; cov:176	chr3	142293004	142293004	C	A	exonic	Gbp2	nonsynonymo	Gbp2:NM_01	14469	GBP2	2634	1	89571815	89616139
T6	VAF:5.69%; cov:123	chr9	54393744	54393744	C	A	exonic	Cib2	nonsynonymo	Cib2:NM_019	56506	CIB2	10518	15	78396948	78423886
T6	VAF:5.71%; cov:140	chr11	51557251	51557251	C	A	exonic	Sec24a	nonsynonymo	Sec24a:NM_0	77371	SEC24A	10802	5	133984479	134063513
T6	VAF:5.71%; cov:140	chr16	20558801	20558801	C	A	exonic	Abcf3	nonsynonymo	Abcf3:NM_01	27406	ABCF3	55324	3	183903811	183911800
T6	VAF:5.71%; cov:140	chr6	71870462	71870462	C	A	exonic	Polr1a	nonsynonymo	Polr1a:NM_0	20019	POLR1A	25885	2	86247339	86333278
T6	VAF:5.76%; cov:139	chr14	55298116	55298116	C	A	exonic	Acin1	nonsynonymo	Acin1:NM_00	56215	ACIN1	22985	14	23527773	23564823
T6	VAF:5.79%; cov:121	chr12	21319702	21319702	C	A	exonic	Cpsf3	nonsynonymo	Cpsf3:NM_01	54451	CPSF3	51692	2	9563697	9613230
T6	VAF:5.79%; cov:121	chr3	90042609	90042609	C	A	exonic	Creb3l4	nonsynonymo	Creb3l4:NM_0	78284	CREB3L4	148327	1	153940010	153946839
T6	VAF:5.83%; cov:120	chr18	67599840	67599840	C	A	exonic	Afg3l2	nonsynonymo	Afg3l2:NM_02	69597	AFG3L2	10939	18	12328943	12377313
T6	VAF:5.88%; cov:119	chrX	6584827	6584827	C	A	exonic	Ccnb3	nonsynonymo	Ccnb3:NM_18	209091	CCNB3	85417	X	49967364	50094909
T6	VAF:5.93%; cov:118	chr7	111380908	111380908	C	A	exonic	Trim6	nonsynonymo	Trim6:NM_00	94088	TRIM6	117854	11	5617339	5634188
T6	VAF:5.97%; cov:134	chr1	167786579	167786579	C	A	exonic	Cd247	nonsynonymo	Cd247:NM_00	12503	CD247	919	1	167399877	167487847
T6	VAF:5.97%; cov:134	chr10	110728549	110728549	C	A	exonic	Osbpl8	nonsynonymo	Osbpl8:NM_0	237542	OSBPL8	114882	12	76745577	76953589
T6	VAF:5.98%; cov:117	chr8	26846538	26846538	C	A	exonic	Ddhd2	nonsynonymo	Ddhd2:NM_02	72108	DDHD2	23259	8	38082736	38133076
T6	VAF:6.14%; cov:114	chr11	102661849	102661849	C	A	exonic	Gjc1	nonsynonymo	Gjc1:NM_001	14615	GJC1	10052	17	42875816	42908184
T6	VAF:6.14%; cov:114	chr2	76551745	76551745	C	A	exonic	Ttn	nonsynonymo	Ttn:NM_0280	22138	TTN	7273	2	179390716	179695529
T6	VAF:6.14%; cov:114	chr3	30996964	30996964	C	A	exonic	Skil	nonsynonymo	Skil:NM_0010	20482	SKIL	6498	3	170075466	170114623
T6	VAF:6.14%; cov:114	chr3	80496307	80496307	C	A	exonic	Gria2	stopgain	Gria2:NM_00	14800	GRIA2	2891	4	158125334	158287227
T6	VAF:6.25%; cov:112	chrX	150124188	150124188	C	A	exonic	Kctd12b	nonsynonymo	Kctd12b:NM_	207474					
T6	VAF:6.35%; cov:126	chr19	9081395	9081395	C	A	exonic	Ahnak	nonsynonymo	Ahnak:NM_00	66395	AHNAK	79026	11	62201016	62323707
T6	VAF:6.36%; cov:110	chr5	129108233	129108233	C	A	exonic	Fzd10	nonsynonymo	Fzd10:NM_17	93897	FZD10	11211	12	130647004	130650285
T6	VAF:6.36%; cov:110	chr5	93594295	93594295	C	A	exonic	Sept11	nonsynonymo	Sept11:NM_001009818:exon8:c.C974A:p.A325E,Sept11:NM_001310669:exon8:c.C974A:p.A325E,Sept11:NM_001310671:exon8:c.C974A:p.A325E,Sept11:NM_001347377:exon8:c.C974A:p.A325E						
T6	VAF:6.48%; cov:108	chr15	7131882	7131882	C	A	exonic	Lifr	nonsynonymo	Lifr:NM_0013	16880	LIFR	3977	5	38475065	38608456
T6	VAF:6.54%; cov:107	chr8	85773457	85773457	C	A	exonic	Tbc1d9	nonsynonymo	Tbc1d9:NM_0	71310	TBC1D9	23158	4	141541919	141677274
T6	VAF:6.56%; cov:122	chr16	38266171	38266171	C	A	exonic	Nr1i2	nonsynonymo	Nr1i2:NM_00	18171	NR1I2	8856	3	119499331	119537332
T6	VAF:6.6%; cov:106	chr5	108641252	108641252	C	A	exonic	Ccdc18	nonsynonymo	Ccdc18:NM_0	73254	CCDC18	343099	1	93645476	93744287
T6	VAF:6.6%; cov:106	chr6	6815183	6815183	C	A	exonic	Dlx6	nonsynonymo	Dlx6:NM_010	13396	DLX6	1750	7	96634860	96640351
T6	VAF:6.64%; cov:286	chr5	14935049	14935049	A	G	exonic	Speer4e	nonsynonymo	Speer4e:NM_	624245					
T6	VAF:6.73%; cov:104	chr2	22433627	22433627	C	A	exonic	Myo3a	nonsynonymo	Myo3a:NM_1	667663	MYO3A	53904	10	26223196	26501456
T6	VAF:6.78%; cov:118	chr14	123906750	123906750	C	A	exonic	Nalcn	nonsynonymo	Nalcn:NM_17	338370	NALCN	259232	13	101706130	102068843
T6	VAF:6.84%; cov:117	chr15	102364831	102364831	C	A	exonic	Atf7	nonsynonymo	Atf7:NM_001	223922	ATF7	11016	12	53901640	54020199
T6	VAF:6.84%; cov:117	chr2	121061970	121061970	C	A	exonic	Trp53bp1	nonsynonymo	Trp53bp1:NM	27223					
T6	VAF:6.96%; cov:115	chr6	90311498	90311498	C	A	exonic	Uroc1	nonsynonymo	Uroc1:NM_00	243537	UROC1	131669	3	126200124	126236616
T6	VAF:7.02%; cov:114	chr7	134940937	134940937	C	A	exonic	Setd1a	nonsynonymo	Setd1a:NM_1	233904	SETD1A	9739	16	30968615	30996437
T6	VAF:7.09%; cov:127	chr2	93038474	93038474	C	A	exonic	Trp53i11	nonsynonymo	Trp53i11:NM_	277414					
T6	VAF:7.14%; cov:112	chr6	55918513	55918513	C	A	exonic	Ccdc129	nonsynonymo	Ccdc129:NM_001081665:exon11:c.C2224A:p.L742I						
T6	VAF:7.27%; cov:110	chr8	74717402	74717402	C	A	exonic	Hsh2d	nonsynonymo	Hsh2d:NM_19	209488	HSH2D	84941	19	16244838	16269386
T6	VAF:7.29%; cov:96	chr17	56141484	56141484	C	A	exonic	Stap2	nonsynonymo	Stap2:NM_14	106766	STAP2	55620	19	4324040	4342783
T6	VAF:7.37%; cov:95	chr13	30978103	30978103	C	A	exonic	Exoc2	nonsynonymo	Exoc2:NM_02	66482	EXOC2	55770	6	485133	693117
T6	VAF:7.37%; cov:95	chr7	25850246	25850246	C	A	exonic	Grik5	nonsynonymo	Grik5:NM_008	14809	GRIK5	2901	19	42502473	42573650
T6	VAF:7.78%; cov:90	chr17	53552973	53552973	C	A	exonic	Efhb	nonsynonymo	Efhb:NM_172	211482	EFHB	151651	3	19920964	19988517
T6	VAF:7.78%; cov:90	chr3	89051033	89051033	C	A	exonic	Krtcap2	nonsynonymo	Krtcap2:NM_0	66059	KRTCAP2	200185	1	155141884	155145951
T6	VAF:7.95%; cov:88	chr6	43235368	43235368	C	A	exonic	Arhgef5	nonsynonymo	Arhgef5:NM_	54324	ARHGEF5	7984	7	144052381	144077725
T6	VAF:8.05%; cov:87	chr3	100877102	100877102	C	A	exonic	Ptgfrn	nonsynonymo	Ptgfrn:NM_01	19221	PTGFRN	5738	1	117452679	117532980
T6	VAF:8.24%; cov:85	chr11	86998495	86998495	C	A	exonic	Trim37	nonsynonymo	Trim37:NM_1	68729	TRIM37	4591	17	57059999	57184282
T6	VAF:8.43%; cov:83	chr8	113116619	113116619	C	A	exonic	Hydin	nonsynonymo	Hydin:NM_17	244653	HYDIN	54768	16	70841281	71264625
T6	VAF:8.54%; cov:82	chr8	89058608	89058608	C	A	exonic	Abcc12	nonsynonymo	Abcc12:NM_1	244562	ABCC12	94160	16	48116884	48189929
T6	VAF:8.74%; cov:103	chr7	148807074	148807074	C	A	exonic	Ap2a2	nonsynonymo	Ap2a2:NM_00	11772	AP2A2	161	11	924894	1012239
T6	VAF:8.86%; cov:79	chr11	72847383	72847383	C	A	exonic	Camkk1	nonsynonymo	Camkk1:NM_0	55984	CAMKK1	84254	17	3763609	3798185
T6	VAF:8.97%; cov:78	chr1	168029936	168029936	C	A	exonic	Dusp27	nonsynonymo	Dusp27:NM_0	240892					
T6	VAF:9.86%; cov:71	chr14	109311398	109311398	C	A	exonic	Slitrk1	nonsynonymo	Slitrk1:NM_19	76965	SLITRK1	114798	13	84451344	84456528
T7	VAF:11.24%; cov:89	chr1	137348841	137348841	T	C	exonic	Nav1	nonsynonymo	Nav1:NM_173	215690	NAV1	89796	1	201592411	201796102
T7	VAF:14.98%; cov:207	chr6	106723462	106723462	T	C	exonic	Trnt1	nonsynonymo	Trnt1:NM_00	70047	TRNT1	51095	3	3168600	3192563
T7	VAF:30.98%; cov:523	chrX	121246299	121246299	T	G	exonic	Vmn2r121;Vmn2r122	nonsynonymo	Vmn2r121:NM	100038941	unkown				
T7	VAF:32.2%; cov:59	chr7	28696650	28696650	T	G	exonic	Zfp974	nonsynonymo	Zfp974:NM_0	73430	unkown				
T7	VAF:58.33%; cov:12	chr4	147048551	147048551	T	C	exonic	Zfp534	nonsynonymo	Zfp534:NM_0	100503584	ZNF616	90317	19	52616344	52643175
T7	VAF:6.73%; cov:223	chr14	31952103	31952103	C	T	exonic	Nt5dc2	nonsynonymo	Nt5dc2:NM_0	70021	NT5DC2	64943	3	52558386	52569070
T7	VAF:6.99%; cov:186	chr17	21366218	21366218	A	-	exonic	Vmn1r234	frameshift del	Vmn1r234:NM	171232	unkown				
T7	VAF:7.26%; cov:124	chr4	115125371	115125371	C	-	exonic	Cyp4a30b	frameshift del	Cyp4a30b:NM	435802	unkown				
T7	VAF:7.56%; cov:119	chr13	26861192	26861192	C	A	exonic	Hdgfl1	nonsynonymo	Hdgfl1:NM_00	15192	unkown				
T7	VAF:8.9%; cov:146	chr19	7032139	7032139	G	C	exonic	Plcb3	nonsynonymo	Plcb3:NM_00	18797	PLCB3	5331	11	64018995	64036622
T8	VAF:13.11%; cov:534	chr1	85153584	85153584	T	A	exonic	Gm38510	nonsynonymo	Gm38510:NM	102638555	unkown				
T8	VAF:23.52%; cov:3070	chr1	87482825	87482825	C	T	exonic	Sp110	nonsynonymo	Sp110:NM_03	109032	SP110	3431	2	231032009	231090444
T8	VAF:35.29%; cov:17	chr5	137633064	137633064	C	T	exonic	Muc3	unknown	UNKNOWN	666339	unkown				
T8	VAF:41.8%; cov:1043	chr7	148824375	148824375	T	G	exonic	Muc6	nonsynonymo	Muc6:NM_00	353328	MUC6	4588	11	1012821	1036706
T8	VAF:5.95%; cov:185	chr1	87506339	87506339	A	C	exonic	Sp140	nonsynonymo	Sp140:NM_00	434484	SP140	11262	2	231067826	231223762
T8	VAF:8.54%; cov:82	chr8	56206165	56206165	T	C	exonic	Gm51573	unknown	UNKNOWN	115486932	unkown				

T9	VAF:11.29%; cov:62	chr8	56205748	56205748	G	-	exonic	Gm51573	unknown	UNKNOWN	115486932	unkown				
T9	VAF:13.33%; cov:75	chr8	57987838	57987838	T	G	exonic	Gm51598	unknown	UNKNOWN	115486972	unkown				
T9	VAF:14.44%; cov:180	chr10	74106317	74106317	G	A	exonic	Pcdh15	unknown	UNKNOWN	11994	PCDH15	65217	10	55562531	57387702
T9	VAF:14.89%; cov:141	chr17	66465019	66465019	G	T	exonic	Washc1	nonsynonymo	Washc1:NM_(	68767	unkown				
T9	VAF:15.87%; cov:63	chr10	41184444	41184444	G	A	exonic	Zbtb24	nonsynonymo	Zbtb24:NM_0	268294	ZBTB24	9841	6	109783797	109804440
T9	VAF:17.24%; cov:58	chr10	75867190	75867190	G	A	exonic	Pcnt	nonsynonymo	Pcnt:NM_001	18541	unkown				
T9	VAF:21.88%; cov:32	chr13	65791780	65791780	T	A	exonic	Gm53057	nonsynonymo	Gm53057:NM	115488140	unkown				
T9	VAF:28.3%; cov:53	chr7	135441575	135441575	C	G	exonic	Rusf1	nonsynonymo	Rusf1:NM_14	233913	unkown				
T9	VAF:6.92%; cov:159	chr14	24205534	24205534	-	T	exonic	Kcnma1	frameshift ins	Kcnma1:NM_(	16531	KCNMA1	3778	10	78629359	79398353
T9	VAF:60%; cov:10	chr7	50445546	50445546	T	C	exonic	Zfp936	nonsynonymo	Zfp936:NM_0	668620	unkown				
T9	VAF:7.48%; cov:254	chr1	85154199	85154199	A	C	exonic	Gm38510	nonsynonymo	Gm38510:NM	102638555	unkown				
T10	VAF:32.43%; cov:37	chr4	147049569	147049569	T	G	exonic	Zfp534	nonsynonymo	Zfp534:NM_0	100503584	ZNF616	90317	19	52616344	52643175
T10	VAF:7.12%; cov:337	chr17	35787388	35787388	A	T	exonic	Sfta2	nonsynonymo	Sfta2:NM_001	433102	unkown				
T10	VAF:9.59%; cov:73	chr8	57987692	57987692	C	G	exonic	Gm51598	unknown	UNKNOWN	115486972	unkown				
TP1	VAF:37.27%; cov:110	chr18	43717880	43717880	G	A	exonic	Jakmip2	stopgain	Jakmip2:NM_(	76217	JAKMIP2	9832	5	146967990	147162338
TP1	VAF:5.36%; cov:168	chr1	40045844	40045846	GAG	-	exonic	Map4k4	nonframeshift	Map4k4:NM_(	26921	MAP4K4	9448	2	102313312	102511149
TP1	VAF:9.64%; cov:197	chr8	15085290	15085290	G	A	exonic	Myom2	nonsynonymo	Myom2:NM_(	17930	MYOM2	9172	HG19_PATCH	1993155	2113475
TP2	VAF:35.78%; cov:218	chr7	26312761	26312761	T	C	exonic	Ceacam2	nonsynonymo	Ceacam2:NM_	26367					
TP2	VAF:39.29%; cov:168	chr5	91517540	91517540	G	A	exonic	Ereg	nonsynonymo	Ereg:NM_007	13874	EREG	2069	4	75230860	75254468
TP3	VAF:25.4%; cov:63	chr6	98966540	98966540	-	G	exonic	Foxp1	frameshift ins	Foxp1:NM_00	108655	FOXP1	27086	3	71003844	71633140
TP3	VAF:27.42%; cov:62	chr6	98966541	98966541	T	C	exonic	Foxp1	nonsynonymo	Foxp1:NM_00	108655	FOXP1	27086	3	71003844	71633140
TP3	VAF:27.54%; cov:138	chr1	74441567	74441567	G	A	exonic	Ctdsp1	nonsynonymo	Ctdsp1:NM_1	227292	CTDSP1	58190	2	219262979	219270664
TP3	VAF:32.45%; cov:151	chr8	108410678	108410678	A	T	exonic	Edc4	nonsynonymo	Edc4:NM_001	234699	EDC4	23644	16	67906926	67918406
TP4	VAF:11.86%; cov:59	chr2	168474067	168474067	T	G	exonic	Atp9a	nonsynonymo	Atp9a:NM_00	11981	ATP9A	10079	20	50213053	50385173
TP4	VAF:18%; cov:100	chr8	57988161	57988161	C	T	exonic	Gm51598	unknown	UNKNOWN	115486972	unkown				
TP4	VAF:24.64%; cov:69	chr6	113445876	113445876	T	G	exonic	Prrt3	nonsynonymo	Prrt3:NM_001	210673	PRRT3	285368	3	9987226	9994078
TP4	VAF:5.04%; cov:238	chr13	104934235	104934235	C	T	exonic	Trappc13	nonsynonymo	Trappc13:NM	66975	TRAPPC13	80006	5	64920543	64962060
TP4	VAF:7.14%; cov:154	chr4	108908338	108908338	-	T	exonic	Calr4	frameshift ins	Calr4:NM_001	108802	unkown				
TP4	VAF:8.44%; cov:225	chr3	89007452	89007452	C	T	exonic	Gba	nonsynonymo	Gba:NM_001(	14466	GBA	2629	HSCHR1_2_CT	155219647	155229865
TP4	VAF:8.91%; cov:101	chr18	63201361	63201361	G	C	exonic	Piezo2	nonsynonymo	Piezo2:NM_0(	667742	PIEZO2	63895	18	10666480	11148587
TP4	VAF:9.09%; cov:99	chr4	131933140	131933140	T	G	exonic	Phactr4	nonsynonymo	Phactr4:NM_(	100169	PHACTR4	65979	1	28696114	28826881
TP5	VAF:14.29%; cov:49	chr8	57987663	57987663	T	A	exonic	Gm51598	unknown	UNKNOWN	115486972	unkown				
TP5	VAF:20.61%; cov:165	chr8	83138189	83138189	T	C	exonic	Frem3	nonsynonymo	Frem3:NM_0(	333315	FREM3	166752	4	144498455	144621828
TP5	VAF:23.33%; cov:60	chr5	123845195	123845195	C	T	exonic	Mlxip	nonsynonymo	Mlxip:NM_13	208104	MLXIP	22877	12	122516628	122631894
TP5	VAF:5.71%; cov:140	chr3	28794605	28794605	A	-	exonic	Gm1527	frameshift del	Gm1527:NM_	385263	unkown				
TP6	VAF:11.11%; cov:135	chr7	116861610	116861610	G	A	exonic	BC051019	nonsynonymo	BC051019:NM	57355	C11orf16	56673	11	8941623	8954553
TP6	VAF:13.21%; cov:53	chr8	56205965	56205965	G	A	exonic	Gm51573	unknown	UNKNOWN	115486932	unkown				
TP6	VAF:14.67%; cov:184	chr5	147294670	147294670	C	T	exonic	Gm6408	nonsynonymo	Gm6408:NM_	623198	unkown				
TP6	VAF:20.56%; cov:214	chr8	63853067	63853067	G	A	exonic	Sh3rf1	nonsynonymo	Sh3rf1:NM_0	59009	SH3RF1	57630	4	170015407	170192256
TP6	VAF:9.17%; cov:109	chr18	42739201	42739201	A	C	exonic	Gpr151	nonsynonymo	Gpr151:NM_1	240239	GPR151	134391	5	145892666	145895753
TP7	VAF:16.23%; cov:1023	chr1	90114824	90114824	C	T	exonic	Ugt1a1;Ugt1a10;Ugt1a2;Ugt	nonsynonymo	Ugt1a8:NM_0	394436	UGT1A1	54658	2	234526291	234681956
TP7	VAF:29.39%; cov:279	chr3	40708872	40708872	A	G	exonic	Abhd18	nonsynonymo	Abhd18:NM_(	269423	unkown				
TP7	VAF:9.64%; cov:83	chr14	102333204	102333204	A	-	exonic	Lmo7	nonframeshift	Lmo7:NM_00	380928	LMO7	4008	13	76194570	76434004
