# Supplementary figures and images for: PUMA-induced apoptosis drives bone marrow failure and genomic instability in telomerase-deficient mice
Source: Cell Death Differ. 2025 Aug 19;33(1):38–50. doi: 10.1038/s41418-025-01557-w (PMC12811261; doi:10.1038/s41418-025-01557-w)

## Slide 1
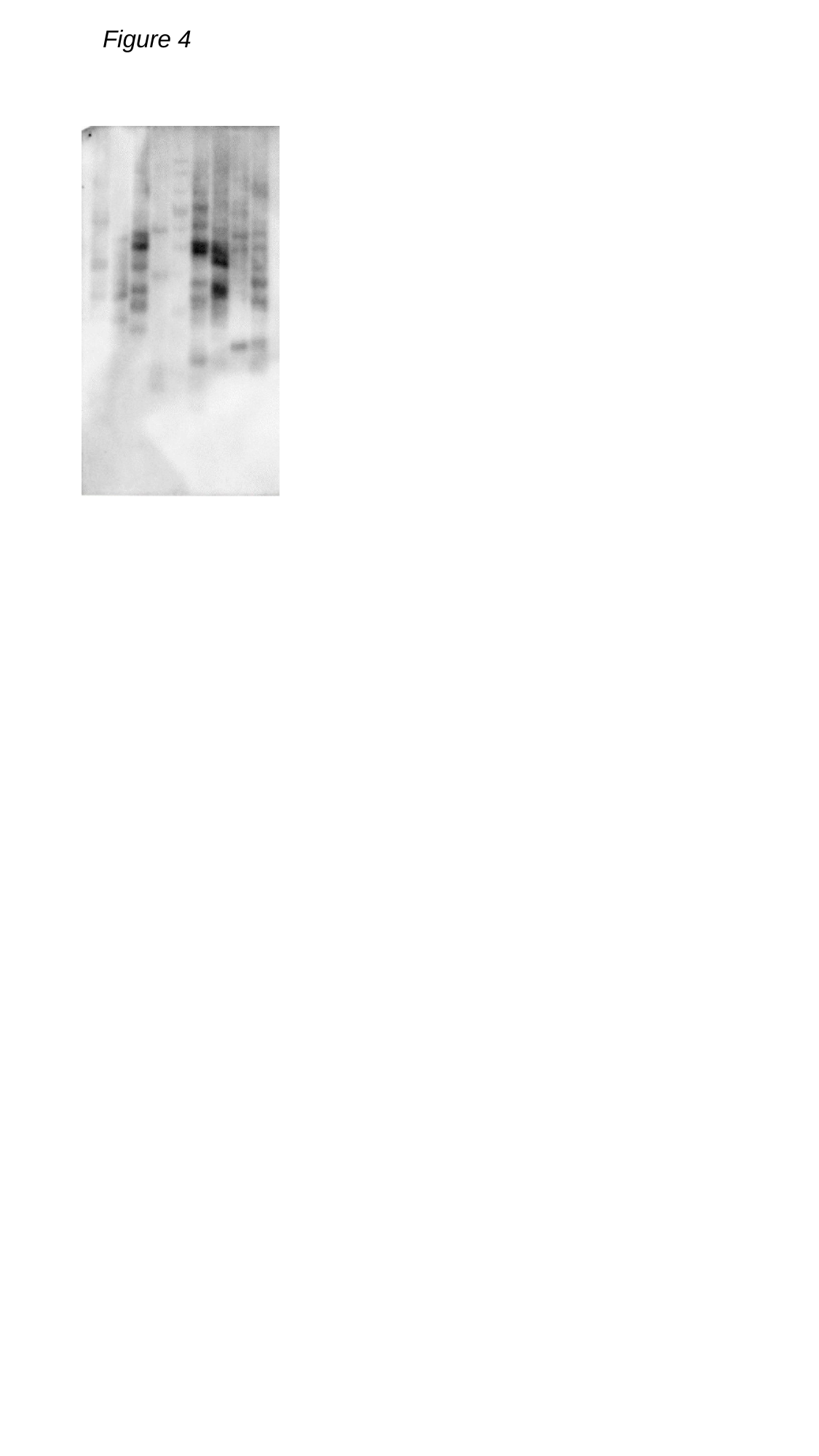

Figure 4

## Slide 2
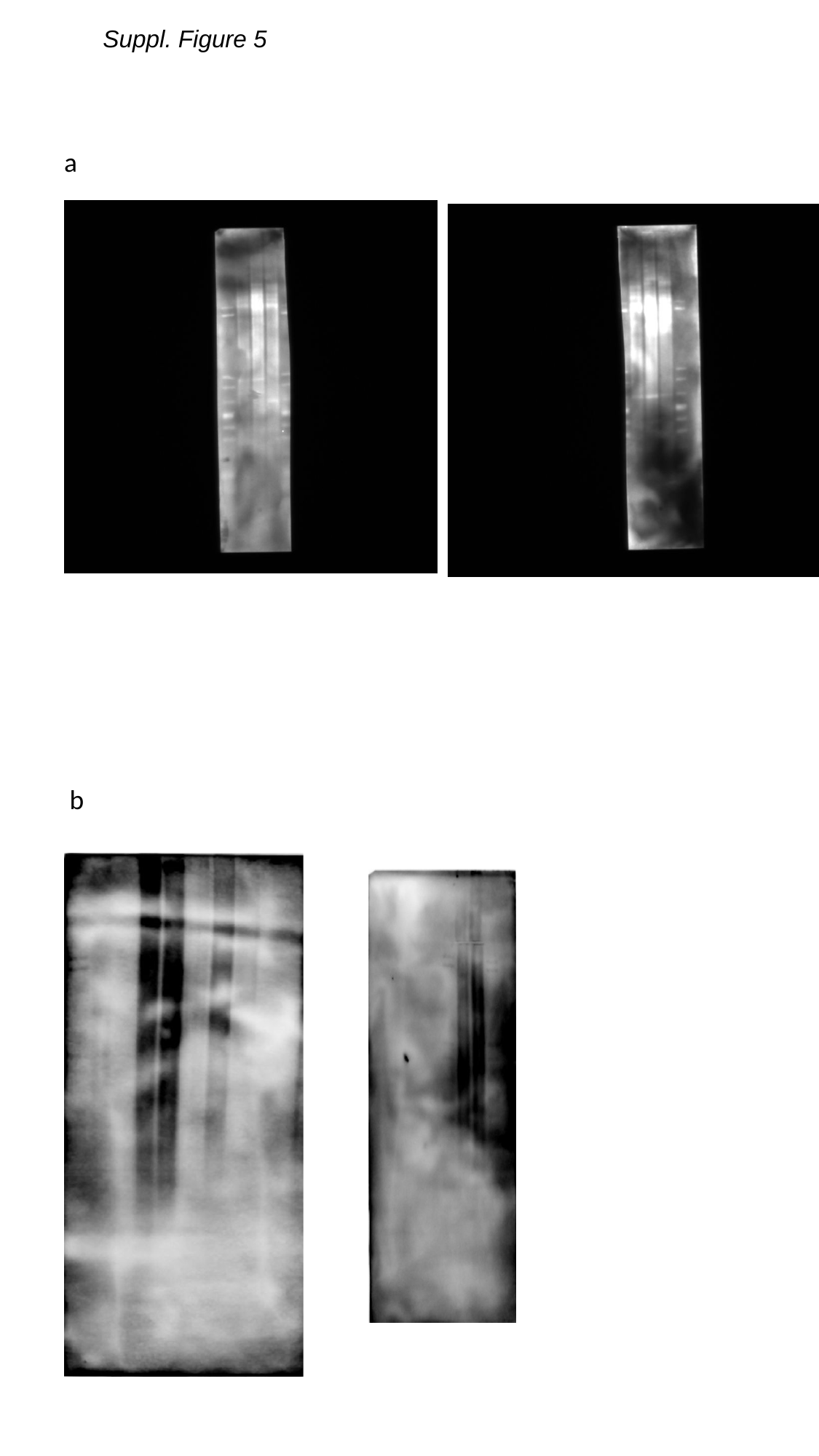

Suppl. Figure 5
a
b

Supplement: Supplementary file 3 — uncropped blots [file 41418_2025_1557_MOESM3_ESM.pptx]
